# Conserved Genes Act as Modifiers of Invertebrate SMN Loss of Function Defects

**DOI:** 10.1371/journal.pgen.1001172

**Published:** 2010-10-28

**Authors:** Maria Dimitriadi, James N. Sleigh, Amy Walker, Howard C. Chang, Anindya Sen, Geetika Kalloo, Jevede Harris, Tom Barsby, Melissa B. Walsh, John S. Satterlee, Chris Li, David Van Vactor, Spyros Artavanis-Tsakonas, Anne C. Hart

**Affiliations:** 1 Department of Neuroscience, Brown University, Providence, Rhode Island, United States of America; 2 Center for Cancer Research, Massachusetts General Hospital, Department of Pathology, Harvard Medical School, Boston, Massachusetts, United States of America; 3 Department of Biology and Biochemistry, University of Bath, Bath, United Kingdom; 4 Department of Cell Biology, Harvard Medical School, Boston, Massachusetts, United States of America; 5 Department of Molecular and Cellular Biology, Brown University, Providence, Rhode Island, United States of America; 6 Department of Biology, City College – City University of New York, New York, New York, United States of America; 7 Collége de France, Paris, France; Stanford University, United States of America

## Abstract

Spinal Muscular Atrophy (SMA) is caused by diminished function of the Survival of Motor Neuron (SMN) protein, but the molecular pathways critical for SMA pathology remain elusive. We have used genetic approaches in invertebrate models to identify conserved SMN loss of function modifier genes. *Drosophila melanogaster* and *Caenorhabditis elegans* each have a single gene encoding a protein orthologous to human SMN; diminished function of these invertebrate genes causes lethality and neuromuscular defects. To find genes that modulate SMN function defects across species, two approaches were used. First, a genome-wide RNAi screen for *C. elegans* SMN modifier genes was undertaken, yielding four genes. Second, we tested the conservation of modifier gene function across species; genes identified in one invertebrate model were tested for function in the other invertebrate model. *Drosophila* orthologs of two genes, which were identified originally in *C. elegans*, modified *Drosophila* SMN loss of function defects. *C. elegans* orthologs of twelve genes, which were originally identified in a previous *Drosophila* screen, modified *C. elegans* SMN loss of function defects. Bioinformatic analysis of the conserved, cross-species, modifier genes suggests that conserved cellular pathways, specifically endocytosis and mRNA regulation, act as critical genetic modifiers of SMN loss of function defects across species.

## Author Summary

Spinal Muscular Atrophy (SMA) is a common, untreatable, and often fatal neuromuscular disease predominately caused by reduced Survival Motor Neuron (SMN) protein function. Here, we use invertebrate models to identify and validate conserved genes that play a critical role in SMN loss of function neuromuscular defects. Decreased SMN function causes growth defects in the nematode *Caenorhabditis elegans* and in the fruit fly *Drosophila melanogaster*—as well as behavioral or synaptic connectivity defects between neurons and muscles, respectively. We found that a genetic modifier of SMA in patients, plastin, also affects SMN function in these invertebrate models. We undertook a genome-wide RNAi screen to identify genes whose perturbation alters the growth defects of *C. elegans* lacking SMN. These genes were validated in neuromuscular assays in nematode and fly models of SMA. Additionally, we used the *C. elegans* model to test SMN modifier genes previously identified in the *Drosophila* SMA model. Combined, these cross-species approaches identified fifteen genes that are important in both species when SMN function is decreased. Related mammalian proteins and the pathways in which they act (including endocytosis and RNA transport/translational control) are likely important players in SMA.

## Introduction

Decreased Survival of Motor Neuron (SMN) protein function underlies most Spinal Muscular Atrophy (SMA) cases [1]. The SMN protein is ubiquitously expressed [2], [3], yet SMA pathology is remarkably specific. Patients lose spinal α-motorneurons and experience muscular dysfunction with atrophy. Mild cases result in slowly progressing muscular weakness, while severe cases dramatically perturb proximal neuromuscular function resulting in childhood death [4]. There is no effective treatment for SMA and at least 1 in 40 people in the US population are carriers of SMN loss of function disease alleles [5]–[7].

The SMN protein is a component of the well-characterized Gemin complex, which assembles splicing machinery in eukaryotes [8]–[10]. SMN also associates with β-actin mRNA during anterograde transport in neuronal processes suggesting a role for SMN in mRNA transport, sub-cellular localization and/or local translation [11]–[15]. In addition, SMN is found in post-synaptic densities and Z-discs of muscles along with other RNA processing proteins [11]–[19]. Roles for SMN in small nucleolar RNA (snoRNA) and microRNA (miRNA) pathways have also been suggested [20]–[22]. The relative contributions of SMN in these various compartments and the relative importance of SMN function in neurons and muscles for SMA pathology have been difficult to determine. Various tissue requirements for SMN function have been observed in different SMA model systems [23]–[27]. The diverse subcellular SMN localization and varied cellular requirements for SMN function suggest that this protein may act in multiple cellular compartments including the neuromuscular junction (NMJ) [28].

To determine in an unbiased fashion which cellular and molecular pathways are particularly relevant to SMA pathology, researchers have turned recently to genetic approaches in vertebrates and invertebrates. The identification of SMN loss of function modifier genes can reveal important biochemical pathways for SMA pathology. Studies in patients have already identified two genes that act as modifiers of SMA: SMN2 and Plastin 3 (PLS3).

Two genes encode human SMN protein: *SMN1* and *SMN2*. The *SMN1* gene encodes only full-length SMN protein while the *SMN2* gene encodes two different transcripts; 10% of *SMN2* transcripts encode a full-length SMN protein identical to the *SMN1* gene product. However, due to a change in the splice consensus sequence, 90% of *SMN2* transcripts contain a stop codon at the beginning of exon 7 and, therefore, encode a truncated protein (called SMNdeltaEx7 or Δ7SMN) of diminished function and stability [1], [29]–[31]. Humans have various numbers of *SMN2* genes; patients with more copies of *SMN2* generally have later onset/less severe symptoms than patients with fewer copies of *SMN2*. Decreased severity and delayed onset is usually attributed to increased full-length SMN levels from *SMN2 in vivo* [17], [32]–[36].

PLS3 may modulate the severity of SMA. In several families, daughters who lack *SMN1* and over-express PLS3 were remarkably unaffected [37]. PLS3 encodes a conserved calcium-binding, actin-bundling/stabilizing protein that is broadly expressed in various tissues including blood, muscles and neurons [38]–[40]. Loss of the yeast PLS3 ortholog, Sac6p, results in defective endocytosis [41], [42]. Altering PLS3 levels modified SMN loss of function defects in zebrafish motorneurons consistent with results in human families and PLS3 co-precipitated with SMN from neuronal tissues [37]. However, increased PLS3 (due to profilin knockdown) did not decrease the defects in an SMA mouse model and it remains unclear how PLS3 might modify SMN neuromuscular defects [43].

Modifier genes identified in patient populations are clearly pertinent to SMA pathology. However, studies in humans are limited by kindred sizes and other considerations. As SMN orthologs are found in *C. elegans* and *Drosophila melanogaster*, it may be more efficient to identify SMA modifier genes in these powerful invertebrate models. SMN loss of function models have already been defined in *C. elegans* and *Drosophila* [18], [25], [44], [45]. Loss of *Drosophila* Smn (*Dm*Smn) causes larval lethality and NMJ defects; *Dm*Smn function is required in neurons and muscles in flies [26]. Loss of *C. elegans* SMN-1 (*Cesmn-1*) also causes neuromuscular function deficits followed by larval lethality [44]. Expression of *Cesmn-1* in neurons dramatically restores neuromuscular function, whereas expression in muscles has little effect [44]. Given SMN conservation across species, genes that act as SMN loss of function modifiers in invertebrates could be important in SMA pathology in humans (e.g. PLS3) [37].

In a recent study, twenty-seven P-element transposon insertion lines were identified in *Drosophila* that modified SMN loss of function defects, and a role for the TGF-beta pathway in SMN loss of function pathology was delineated [26]. However, it remains unclear for several P-element lines which *Drosophila* gene near the transposon insertion site is responsible for modulating SMN phenotypic defects. The *Drosophila* P-element lines carried an inducible GAL4-UAS that could drive either over-expression or antisense RNAi expression of neighboring genes depending on transposon insertion site. Additionally, insertion of the P-element itself might perturb gene function. Eliminating ambiguity regarding modifier gene identity would increase the utility of the *Drosophila* study.

To explore the genetic circuitry affecting SMN activity in *C. elegans*, the *Cesmn-1(lf)* growth defect phenotype was used as a metric in a rapid large-scale genetic screen. Growth may be affected by a variety of changes, such as body length and longevity. Subsequently, modifier genes were tested using a *C. elegans* behavioral assay, the pharyngeal pumping, which is likely more pertinent to SMN loss of function neuromuscular defects. In addition, to identify conserved invertebrate SMN modifier genes, we utilized previously described *Drosophila* assays to assess genetic interaction of *DmSmn* with *Drosophila* orthologs of *C. elegans* modifier genes. In the study by Chang and co-workers, the *Dm*Smn lethal phenotype correlated with NMJ defects for virtually all *Dm*Smn modifier genes, suggesting that lethality and neuromuscular bouton number are effective measures of genetic interaction with the *Drosophila* SMN ortholog [26].

Here, we define conserved genetic modifiers of SMN loss of function using *C. elegans* and *Drosophila*. We find that PLS3 orthologs act as SMN modifier genes in both invertebrate species. A genome-wide RNAi screen in *C. elegans* identified four new SMN modifier genes, including *ncbp-2* and *flp-4*, which also modify SMN loss of function defects in *Drosophila*. Candidate SMN modifier genes identified in a previous *Drosophila* screen were tested in *C. elegans* yielding twelve cross-species modifier genes. Examination of the literature for these genes suggested specific cellular pathways that are critical genetic modifiers of SMN function: endocytosis and RNA processing. These pathways may also be pertinent to SMN loss of function defects in patients with SMA.

## Results

The previously described *Cesmn-1(ok355)* deletion allele causes a complete loss of *Cesmn-1* function and is referred to herein as *Cesmn-1(lf)* [44]. *Cesmn-1(lf)* is recessive; heterozygous animals are overtly normal. To facilitate identification of heterozygous *versus* homozygous animals, we utilized the balanced strain *hT2(bli-4(e937) let-?(q782) qIs48[myo-2p::GFP])/Cesmn-1(lf)* (abbreviated *+/Cesmn-1(lf)* [44], [46]. Heterozygous *+/Cesmn-1(lf)* animals express pharyngeal GFP, homozygous *Cesmn-1(lf)* progeny do not express GFP, and progeny homozygous for the *hT2* balancer die as GFP-expressing embryos.

**Figure 1:**
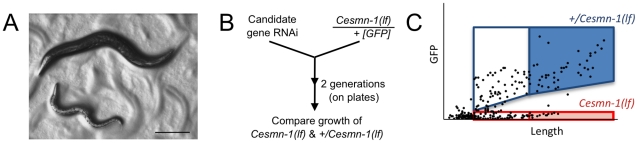
Survival and average length of *Cesmn-1(lf)* animals is decreased. Homozygous loss of *Cesmn-1(lf)* results in slower growth and most animals die during larval stages. Animals that reach the adult stage are short-lived and sterile. A) Image of age-matched heterozygous *hT2(lethal)[myo-2p::GFP]/Cesmn-1(lf)* (*+/Cesmn-1(lf)*, described in text) and homozygous *Cesmn-1(lf)* individuals (below). Scale bar indicates 150 microns. B) To test the impact of candidate modifier genes on growth rates and size, a *C. elegans* growth assay was established. *+/Cesmn-1(lf)* animals were collected as unhatched eggs, reared for 5 days/2 generations on standard *C. elegans* plates upon RNAi bacterial strains, as illustrated in the flow chart. Bacterial cultures expressed dsRNA corresponding to the gene of interest for RNAi knockdown; bacterial cultures containing vector with no insert were used as a control (‘empty’). C) Using an automated system, the length and fluorescence of animals was determined [51]; the smallest larvae in each culture were indistinguishable from debris and were excluded from the analysis as illustrated in the graph. Genotypes were discriminated by GFP fluorescence and length was determined as ‘time-of-flight’ through the laser chamber; each dot in the graph represents an individual animal. The disparate growth/survival rates of *Cesmn-1(lf)* and *+/Cesmn-1(lf)* animals alters the percentage of ‘large’ animals in these mixed stage cultures. As illustrated graphically, *+/Cesmn-1(lf)* and *Cesmn-1(lf)* animals can be sorted into blue and red boxes, respectively; animals designated as ‘large’ fall into shaded boxes. The fraction of large animals was calculated (# animals in shaded box/# animals in both shaded and unshaded box for each genotype) and is reported as % large with standard error of the mean (S.E.M.); significance was determined by Mann-Whitney *U* two-tailed test with p≤0.05. At least three independent cultures of more than 200 animals were scored for each targeted gene.

Although complete loss of SMN function causes lethality, *C. elegans* that are homozygous mutant for *Cesmn-1(lf)* can survive for several days due to partial maternal rescue. It has been suggested that *+/Cesmn-1(lf)* hermaphrodites load sufficient *Cesmn-1* maternal protein and/or perhaps mRNA into oocytes to support development through embryogenesis and early larval stages [44]. Accordingly, homozygous *Cesmn-1(lf)* larvae initially resemble wild type animals. Eventually maternally-loaded *Cesmn-1* product is lost; *Cesmn-1(lf)* animals grow more slowly than *+/Cesmn-1(lf)* siblings, are shorter, sterile, and most *Cesmn-1(lf)* animals die before reaching adulthood (Figure 1A). Combined, these defects decrease the average size of the *Cesmn-1(lf)* population *versus* control animals; decreased average population size will be referred to herein as a growth defect. This growth defect was harnessed in an automated assay to identify *Cesmn-1(lf)* modifier genes in a genome-wide screen.

### *C. elegans* growth assay

To validate growth as an assay for SMN modifier gene identification, we first demonstrated that RNAi knockdown of *Cesmn-1* or the invertebrate ortholog of Plastin 3 (PLS3) altered *Cesmn-1(lf)* growth. The *C. elegans* gene *plst-1* (*PLaSTin (actin bundling protein) homolog-1*) encodes a predicted protein similar to PLS3.

To knockdown gene function, *C. elegans* were reared on bacteria producing double stranded RNA corresponding to the gene of interest, a strategy known as ‘feeding RNAi’ [47]. Feeding RNAi decreases gene transcripts in most *C. elegans* tissues although knockdown in neurons is generally less effective than knockdown in muscles, germline, and other tissues [48]–[50]. Here, animals were reared for two generations on solid media and RNAi feeding bacteria corresponding to *Cesmn-1* or *plst-1*, allowing knockdown of maternal and zygotic transcripts (Figure 1B). Bacteria containing the empty RNAi feeding vector were used as a negative control (*empty(RNAi)*).

An automated system was used to simultaneously measure growth and determine genotype for the progeny o*f +/Cesmn-1(lf)* animals (Figure 1C). The COPAS BioSorter (Union Biometrica, Holliston, MA) measures *C. elegans* length as ‘time-of-flight’, which is the time required for the animal to pass through the fluorescence-detection chamber [51]. *Cesmn-1(lf)* homozygous animals do not express GFP while *+/Cesmn-1(lf)* heterozygous animals express GFP and are longer than *Cesmn-1* homozygous animals of the same late larval or adult stage. Animals smaller than the L2 larval stage were excluded from this analysis to avoid bacterial debris. The percentage of large adult animals was determined for each genotype and RNAi treatment.

RNAi knockdown of *Cesmn-1* decreased the percentage of large animals in both *Cesmn-1(lf)* homozygous and +/*Cesmn-1(lf)* heterozygous populations (Table 1, Rows 1 & 2). Initially, it seems counter-intuitive that the defects of *Cesmn-1(lf)* animals are exacerbated by *Cesmn-1(RNAi)*. However, in this scenario, transcripts in both the somatic tissues and germline of *+/Cesmn-1(lf)* heterozygous animals are targeted and, consequently, maternally-loaded *Cesmn-1* transcript and protein are depleted in homozygous *Cesmn-1(lf)* progeny, abrogating partially the observed maternal rescue. The ability of *Cesmn-1(RNAi)* to exacerbate *Cesmn-1(lf)* defects suggests that the effects of modifier genes can be assessed using RNAi feeding.

**Table 1:** Conserved genes modify *Cesmn-1(lf)* defects in the growth assay.

*Ce* gene	*Dm* gene	*Cesmn-1(lf)*	*+/Cesmn-1(lf)*
		% large ± SEM	% large ± SEM
none	none	18±2	46±1
*Cesmn-1*	Smn	6±4*	31±1*
*plst-1*	Fimbrin	15±3	55±3*
*uso-1*	p115	7±4*	44±1
*nhr-85*	Eip75B	8±5*	44±1
*egl-15*	Breathless	9±5*	45±1
*atf-6*	Atf6	11±7*	40±4
*ape-1*	CG18375	11±9*	50±2
*kcnl-2*	SK	12±1*	42±1
*nekl-3*	Nek2	13±3*	43±2
*atn-1*	Actinin	23±4*	43±1
*cash-1*	CG33172	26±5	64±11*
*dlc-1*	cut up	15±8	66±3*

*C. elegans* modifier genes are listed in column 1 and orthologous *Drosophila* genes are listed in column 2. Only genes that significantly altered the percentage of large *Cesmn-1(lf)* homozygous animals (column 3) or *+/Cesmn-1(lf)* heterozygous animals (column 4) are presented. Animals were reared for two generations on bacterial RNAi strains targeting the *C. elegans* gene prior to growth analysis as described in Figure 1. The percentage of large animals was determined for each genotype after RNAi knockdown of the indicated *C. elegans* gene and is reported with standard error of the mean (S.E.M.). Large animals include adults, L4 and L3 stage larvae. This assay does not distinguish between slow growth, changes in relative length, and altered viability. Three to six independent determinations were undertaken for each genotype/RNAi culture and the percentage of large animals in each trial was averaged. Significant changes from *empty(RNAi)* for each RNAi/genotype using the two-tailed Mann-Whitney *U* test (p<0.05) are indicated with an asterisk. See Table S1 for results for all genes tested and Materials and Methods for details.

Knockdown of the *C. elegans* PLS3 ortholog, *plst-1*, increased the average length of the *+/Cesmn-1(lf)* population, but did not significantly alter the average length of *Cesmn-1(lf)* animals (Table 1, Rows 1 & 3). Genetic interaction with *plst-1* was further confirmed by using the *plst-1 (tm4255)* mutant allele (Table 2). The average length of *+/Cesmn-1(lf);plst-1(tm4255)* adult animals was significantly increased in relation to *+/Cesmn-1(lf)* animals. In contrast, the average length of homozygous *Cesmn-1(lf);plst-1(tm4255)* was not altered, recapitulating the results of *plst-1(RNAi)*. Increased average adult length is an overall growth metric thzat may encompass a variety of changes; decreased *plst-1* function, by RNAi or mutant allele, could increase length, cause sterility, and/or increase longevity in +/*Cesmn-1(lf)* control animals. It appears that loss of *Cesmn-1* function suppresses the effects of decreased *plst-1* function (*i.e.* increased length was not observed in *Cesmn-1(lf);plst-1(tm4255)* homozygous mutant animals). The genetic and functional relationship between SMN and PLS3 bears further examination; as *plst-1* and *Cesmn-1* have opposing effects on the growth assay and since *Cesmn-1(lf);plst-1(tm4255)* animals resemble *Cesmn-1(lf)* single mutants, *Cesmn-1* may act downstream of *plst-1* in this growth assay [52].

**Table 2:** *C. elegans* orthologs of PLS3, GRK, and FMRFamide modify *Cesmn-1(lf)* growth defects.

*Ce* genotype	*Ce* gene	*Cesmn-1(lf)*	*+/Cesmn-1(lf))*
	*RNAi target*	% large ± SEM	% large ± SEM
	none	27±2	44±2
*plst-1(tm4255)*	none	39±5	67±2*
*grk-2(rt97)*	none	10±1*	41±1
*flp-4(yn35)*	none	20±2	51±2

The growth defects of +/*Cesmn-1(lf)* and *Cesmn-1(lf)* animals were examined in the presence of *grk-2(rt97)*, *flp-4(yn35)* and *plst-1(tm4255)* loss of function alleles; alleles for each modifer gene are listed in column 1. The percentage of large animals was determined for *Cesmn-1(lf)* homozygous animals (column 3) or *+/Cesmn-1(lf)* heterozygous animals (column 4). At least three independent determinations were undertaken for each genotype. Significance *versus* control genotypes (*+/Cesmn-1(lf)* and *Cesmn-1(lf)*) was determined using Chi-square analysis (p<0.05 indicated with an asterisk). S.E.M. is also reported.

### Growth modifier genes identified in *C. elegans* genome-wide screen

**Figure 2:**
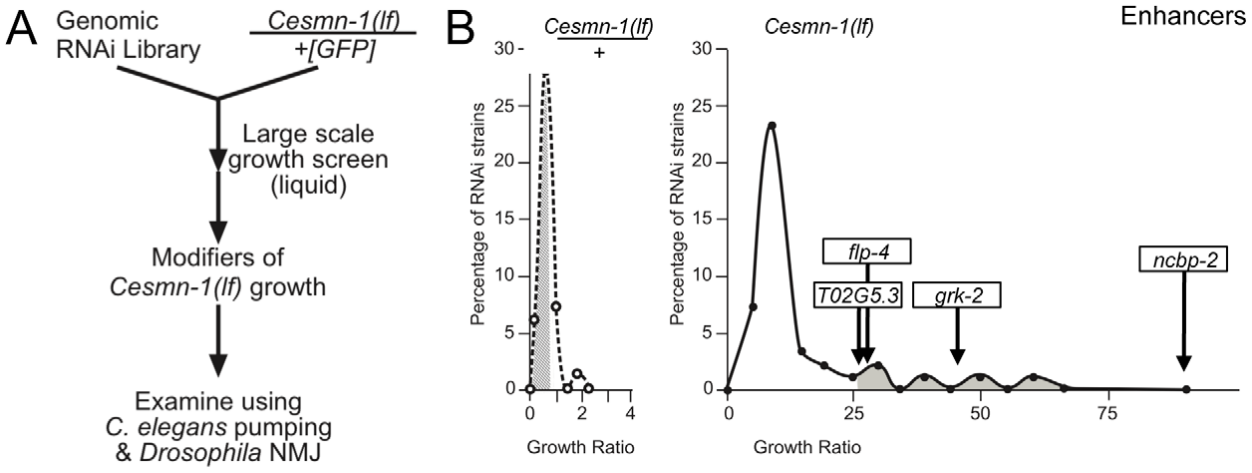
Genome-wide RNAi screen for *Cesmn-1(lf)* modifier genes. A) To identify genes whose knockdown modifies the growth of *Cesmn-1(lf)* animals, the progeny of *hT2(lethal)[myo-2p::GFP]/Cesmn-1(lf)* (*+/Cesmn-1(lf)*, described in text) were reared for more than 2 generations in 96-well, liquid culture format on bacteria expressing dsRNA corresponding to over 16,500 *C. elegans* genes. Modifier genes identified in this assay were tested subsequently in neuromuscular assays in *C. elegans* and *Drosophila*. B) *Cesmn-1(lf)* and *+/Cesmn-1(lf)* length was measured for each RNAi clone and a ‘growth’ ratio of large∶small animals was determined for each genotype. Representative graphs illustrate the distribution of RNAi clone growth ratios. Candidate enhancer genes were those with a growth ratio more than 2 standard deviations above the mean for *Cesmn-1(lf)* (shaded in right graph) and within 0.7 standard deviations of the mean for *+/Cesmn-1(lf)* (shaded in left graph) for each 96-well plate. No suppressors were identified using similar criteria. Two independent determinations were made for each clone in the original screen. Candidate genes were retested in at least quadruplicate and enhanced growth in at least 40% of trials before designation as growth modifier genes; average growth ratios of enhancers from the *C. elegans* screen are indicated.

To identify additional genes that modify SMN loss of function defects, a large-scale genome-wide screen for enhancers and suppressors of the *Cesmn-1(lf)* growth defect was undertaken. The growth assay was adapted to a higher-throughput 96-well, liquid culture format and a previously described genome-wide *C. elegans* RNAi feeding library was used for gene knockdown (Figure 2A) [53]. Progeny of *+/Cesmn-1(lf)* animals were reared for two weeks (more than 2 generations) on RNAi feeding bacterial strains before assessment of growth using the COPAS Biosorter [51]. To identify RNAi clones that specifically altered the growth of *Cesmn-1(lf)* animals, a growth ratio of large to small animals was determined for each clone for *Cesmn-1(lf)* and for *+/Cesmn-1(lf)* genotypes. If the RNAi clone growth ratio was more than 2 standard deviations away from the mean for *Cesmn-1(lf)* animals and within 0.7 standard deviations of the mean for *+/Cesmn-1(lf)* animals in at least 40% of independent trials, then the corresponding gene was designated as an *Cesmn-1(lf)* modifier (Figure 2B). In the primary high-throughput screen, no suppressors were found, but four genes were identified as enhancers (Figure 2B). RNAi knockdown of these genes exacerbated homozygous *Cesmn-1(lf)* growth defects and did not significantly alter the growth of heterozygous *+/Cesmn-1(lf)* animals: *ncbp-2*, *T02G5.3*, *grk-2*, and *flp-4*. *ncbp-2* encodes the *C. elegans* Cap Binding Protein 20 (CBP20 or Cbp20) ortholog [54]. *T02G5.3* encodes a predicted protein of unknown function with no vertebrate orthologs based on BLAST analysis. *grk-2* encodes one of two G-protein coupled receptor kinases. *flp-4* encodes an FMRFamide family neuropeptide protein. The low number of modifiers identified in this screen versus the previous *Drosophila* screen may reflect the stringent criterion utilized here or the inefficiency of RNAi by feeding in neurons.

To determine if decreased adult body length accounts for the enhanced *Cesmn-1(lf)* growth defect upon knockdown of *ncbp-2*, *T02G5.3*, *grk-2* and *flp-4*, the average body length of *Cesmn-1(lf)* young animals was determined (Text S1 and Table S4, top panel). Only *ncbp-2(RNAi)* significantly reduced the average body length of *Cesmn-1(lf)* animals suggesting that the enhanced growth defect caused by *ncbp-2(RNAi)* could be attributed to the *Cesmn-1(lf)* shorter body size. The other three enhancer genes may alter survival or growth as adult animals.

SMA is a neuromuscular disease and, therefore, our objective was the identification of modifier genes that impact SMN neuromuscular function. We then examined the impact of *Cesmn-1* growth modifier genes on *Cesmn-1* loss of function neuromuscular defects using RNAi and, when available, loss of function alleles of modifier genes.

### RNAi knockdown of *ncbp-2*, *grk-2*, and *T02G5.3* modified *Cesmn-1(lf)* neuromuscular defects

**Figure 3:**
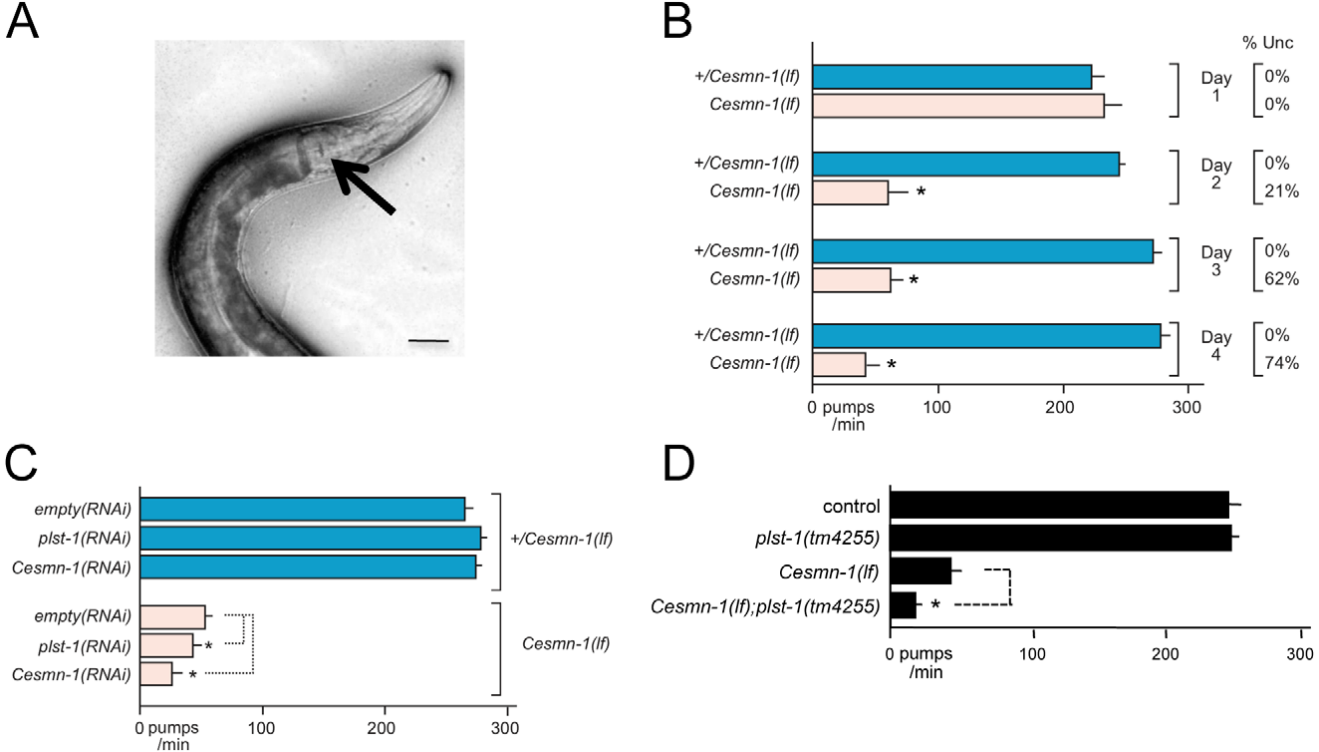
Loss of *C. elegans* PLS3 ortholog enhances the pharyngeal pumping defects of *Cesmn-1(lf)* animals. During feeding the *C. elegans* pharynx contracts at over 200 times per minute to capture and grind bacteria. A) Pharyngeal pumping rates can be determined by videotaping feeding and counting contractions as movement of the grinder (indicated by arrow) at slower playback speeds; scale bar indicates 40 microns. B) The progeny of *hT2(lethal)[myo-2p::GFP]/Cesmn-1(lf)* (*+/Cesmn-1(lf)*) animals were allowed to hatch on bacterial strains on standard *C. elegans* plates. Pumping rates of *+/Cesmn-1(lf)* heterozygous and *Cesmn-1(lf)* homozygous animals were determined on different days (day 1–4) on a control ‘empty vector’ bacterial strain. Decreased locomotion (sinusoidal movement) was also scored as uncoordinated (Unc). At day 1, the pumping rates and locomotion of *Cesmn-1(lf)* animals are identical to *+/Cesmn-1(lf)* or wild type animals. By day 2, the average pumping rates dropped dramatically (as previously reported [44]) and roughly 20% of animals were uncoordinated (column on right). By day 3 and 4, the pumping rates and locomotion of the majority of surviving animals are defective. Expressing *Cesmn-1* in neurons is sufficient to restore pumping rates to near normal levels [44]. The behavior of *Cesmn-1(lf)* animals is initially normal due to maternal loading of *Cesmn-1* gene products, but as the maternal contribution is depleted, loss of *Cesmn-1* causes progressive defects in neuromuscular function. C) Pumping rates of *+/Cesmn-1(lf)* and *Cesmn-1(lf)* animals were determined at 3 days post-hatching on bacterial feeding strains expressing dsRNA corresponding to *Cesmn-1* and *plst-1* genes; ‘empty vector’ bacterial cultures were used as a control (‘empty’). *plst-1(RNAi*) or *Cesmn-1(RNAi)* significantly descreased *Cesmn-1(lf)* pumping rates, but not *+/Cesmn-1(lf)* pharyngeal pumps. D) Decreasing *plst-1* function has no effect on pumping rates in heterozygous *+/Cesmn-1(lf)* animals, but pumping rates were significantly decreased in *Cesmn-1(lf); plst-1(tm4255)* double mutant animals. At least 20 animals were scored for each genotype in at least 2 independent trials. Pumping rates reported are the average of all animals in all trials. Because of the known impact of chromosomal pairing on histone methylation and gene expression [96], all animals tested herein were the progeny of animals carrying the *hT2* balancer chromosome that prevents recombination. S.E.M. is reported. Significance of p≤0.05 is indicated with an asterisk and was determined using either an unpaired two-sample *t*-test or a Mann-Whitney *U* two-tailed test according to sample-specific parameters [100].

A recent study from the Sattelle laboratory demonstrated that loss of *Cesmn-1* function causes progressive defects in *C. elegans* neuromuscular function in pharyngeal pumping [44]. *C. elegans* feeds on bacteria and other microorganisms using a small, discrete subset of neurons and muscles contained in the pharynx (Figure 3A) [55]. Pharyngeal cell specification, neuronal development, and myoblast fusion is completed within hours of hatching [56], [57]. The pharynx pumps continuously and symmetrically at over 250 beats per minute in wild type animals when food is present and larval pumping is interrupted only by molting under standard culture conditions. We confirmed a previous report [44] that in early larval stages, the pumping rates of *Cesmn-1(lf)* animals are indistinguishable from control animals, but at later larval stages *Cesmn-1(lf)* pumping rates drop (Figure 3B).*Cesmn-1(lf)* animals have progressive defects in pharyngeal pumping, which occur earlier than reported locomotion defects. At day 2, 62% of *Cesmn-1(lf)* animals are moving spontaneously, but pumping rates have dropped dramatically (Figure 3B). Restoration of *Cesmn-1* function in neurons almost completely restores pumping rates suggesting that *Cesmn-1* is required in neurons for this behavior [44].

The efficacy of RNAi by feeding in this neuromuscular assay was assessed for *Cesmn-1* and *plst-1* using *Cesmn-1(lf)* and *+/Cesmn-1(lf)* animals. Animals were allowed to hatch on RNAi feeding plates and pumping rates were determined after three days. Either *plst-1(RNAi)* or *Cesmn-1(RNAi)* decreased *Cesmn-1(lf)* pumping rates, but not *+/Cesmn-1(lf)* pumping rates (Figure 3C). In addition, *plst-1(lf)* significantly decreased the pumping rates of *Cesmn-1(lf)* animals, validating the genetic interaction of *plst-1* with *Cesmn-1* in the neuromuscular pharyngeal pumping assay (Figure 3D). This exacerbation of *Cesmn-1* loss of function defects by *plst-1* manipulation is consistent with results in other organisms [37]. The ability of *Cesmn-1(RNAi)* and *plst-1(RNAi)* to alter pumping of homozygous mutant *Cesmn-1(lf)* animals suggests that candidate modifier genes can be assessed using RNAi knockdown in this neuromuscular assay.

**Figure 4:**
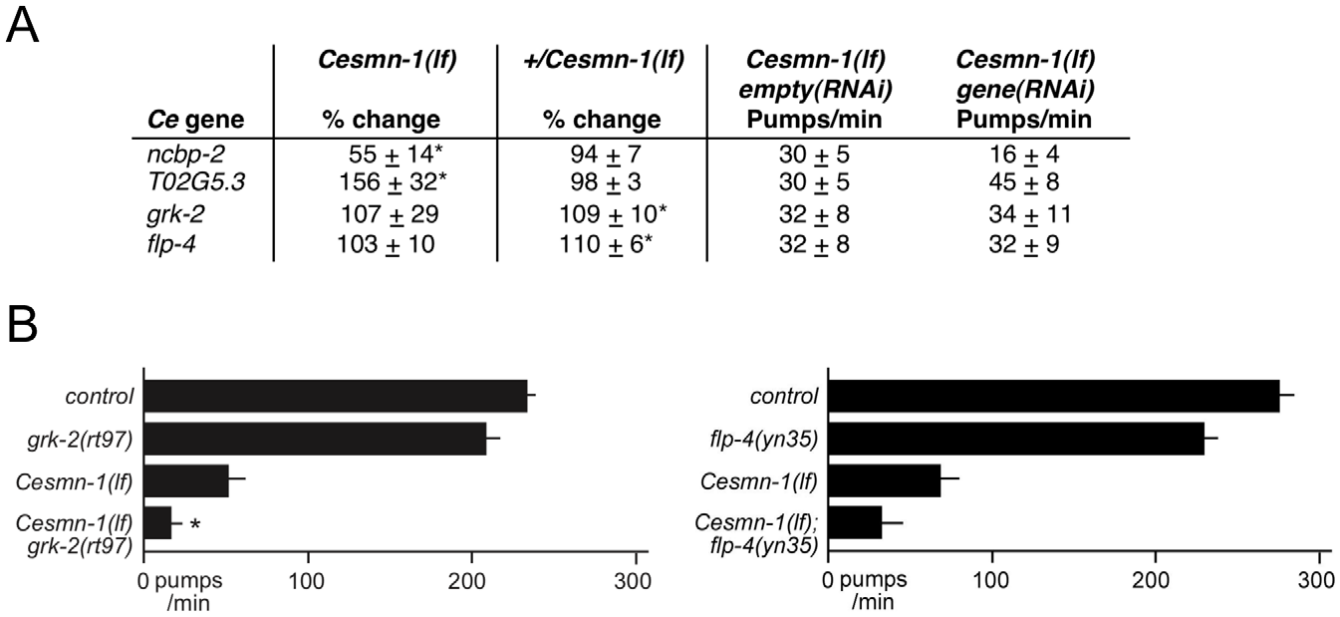
*C. elegans* RNAi screen identifies conserved SMN modifier genes. A) Four genes were identified as modifiers of *Cesmn-1(lf)* growth defects in the *C. elegans* RNAi screen: *ncbp-2*, *T02G5.3*, *grk-2* and *flp-4*. The pharyngeal pumping rates of homozygous mutant *Cesmn-1(lf)* and heterozygous control *+/Cesmn-1(lf)* animals were determined at day 3 as described in Figure 3. B) For two *C. elegans* candidate modifier genes, *flp-4* and *grk-2*, loss of function alleles were available. *grk-2(rt97)* significantly decreased the pumping rates of *Cesmn-1(lf)* animals, while *flp-4(yn35)* lowered *Cesmn-1(lf)* pumping rates, but did not reach statistical significance (p = 0.012 and p = 0.236 by two-tailed Mann-Whitney *U* test, respectively). Pumping rates were determined on day 3 post-hatching; at least 25 animals were scored for each genotype in at least 3 independent trials. Pumping rates reported are the average of all animals in all trials. Significance *versus Cesmn-1(lf)* is indicated with an asterisk. To control for genetic background, all animals tested were the progeny of animals carrying the balancer chromosome *hT2[myo-2p::GFP]*; control animals are *hT2/Cesmn-1(lf)* siblings.

The four modifier genes from the *C. elegans* growth screen were tested for function as *Cesmn-1* neuromuscular modifier genes using the pharyngeal pumping assay. Results are summarized in Figure 4A. *ncbp-2(RNAi)* and *T02G5.3(RNAi)* enhanced and suppressed the pharyngeal pumping defects of *Cesmn-1(lf)* animals, respectively; *flp-4(RNAi)* and *grk-2(RNAi)* had no significant effect compared to controls. We suggest that *ncbp-2* and *T02G5.3* are likely modifiers of *Cesmn-1(lf)* neuromuscular defects based on RNAi results.

### Loss of *grk-2* enhances *Cesmn-1(lf)* growth and neuromuscular defects

RNAi knockdown of *C. elegans* genes by feeding is robust in virtually all cell types but can often be inefficient and can result in only partial loss of gene function, especially in the nervous system [58], [59]. To address the specificity of the genetic modifiers, the RNAi results were confirmed by using mutant alleles when possible; alleles of *ncbp-2* and *T02G5.3* were not available.

A *grk-2* loss of function allele has been previously described, *grk-2(rt97)* [60]. Loss of *grk-2* significantly enhanced the growth defects of *Cesmn-1(lf*) animals (Table 2). Additionally, the pumping rates of *grk-2(lf)* animals derived from *hT2* parents were not significantly lower than those of control animals, but the average pumping rates of *Cesmn-1(lf) grk-2(lf)* double mutant animals were significantly lower than the pumping rates of either single mutant (Figure 4B). This suggests that *grk-2* loss enhances both *Cesmn-1(lf)* growth and pharyngeal pumping defects. A *grk-2* gain of function allele is not available and transgenes are unstable in *+/Cesmn-1(lf)* animals (unpublished results and [44]).

To test the genetic interaction of *flp-4* with *Cesmn-1*, we identified a *flp-4* loss of function allele, *flp-4(yn35)*, using PCR based screening techniques [61]–[65]. The *flp-4(yn35)* deletion removes all sequences encoding FLP-4 FMRFamide neuropeptides and likely causes a complete loss of *flp-4* function ([66] and C. Li, *in preparation*). Although *flp-4(yn35)* reduced the percentage of *Cesmn-1(lf)* large animals in the growth assay, the difference was not statistical significant different (Table 2). Similar results were obtained using the pharyngeal pumping assay. The pumping rates of *flp-4(lf)* animals were slightly lower, but not significantly different than control animals. Loss of *flp-4* function decreased pumping rates of *Cesmn-1(lf)* animals in five independent trials, but the difference was not statistically significant (p = 0.236, Figure 4B). Either *flp-4* is not a *bona fide* modifier or *flp-4(RNAi)* may act off-target decreasing the function of more than one of the 32 other *C. elegans* FMRFamide genes [67].

### *Drosophila* orthologs of *plst-1*, *ncbp-2* and *flp-4* modify SMN neuromuscular defects

SMN modifier genes that are conserved across species would be of considerable interest. Three of the candidate genes identified in the *C. elegans* screen encode conserved proteins with clear orthologs in other species: *grk-2*, *flp-4*, and *ncbp-2*. To determine if their orthologs modify SMN loss of function defects, we turned to the fruit fly *Drosophila*. Decreased function in the *Drosophila* SMN ortholog Smn (*Dm*Smn) results in growth defects, early pupal arrest, and NMJ synaptic defects [26]. We utilized pre-existing *Drosophila* loss of function alleles and previously described *Drosophila* assays to assess genetic interaction of *Dm*Smn with *Drosophila* orthologs of *C. elegans* modifier genes [18], [25], [26].

**Figure 5:**
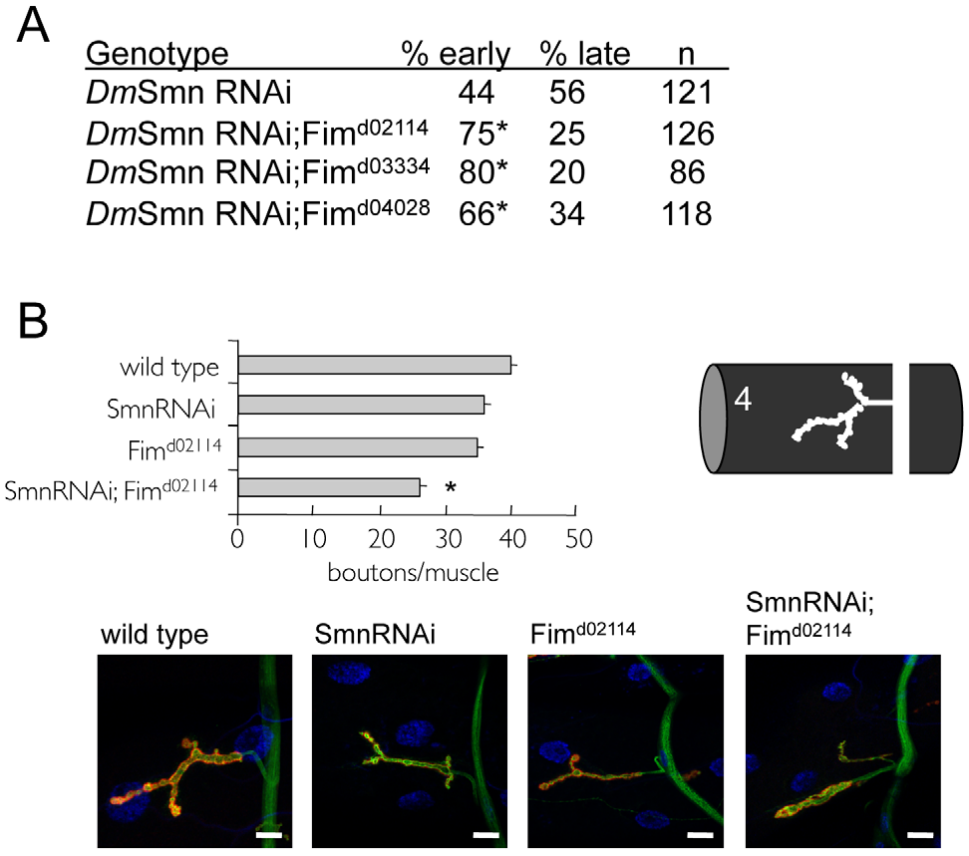
Loss of PLS3 orthologs enhances SMN loss of function defects in invertebrates. Previous studies in vertebrates suggested that PLS3 orthologs in *C. elegans* and *Drosophila* might modify SMN loss of function defects [37]. A) A genetic interaction was found between the *Drosophila* PLS3 ortholog (Fimbrin or Fim) and *Dm*Smn using previously described *Dm*Smn RNAi knockdown lines [26] and Fim loss of function alleles. Percentage early or late larval lethality is reported. Ubiquitous RNAi knockdown of *Dm*Smn using the *tubulinGAL4* (TubGAL4) driver results in pupal death; modifier genes alter the percentage of animals that die at early *versus* late pupal stages (day 7 versus day 9 [26]). Loss of Fim function significantly increased the percentage of animals that died as early pupae (p≤0.05 by Chi-square analysis); Fim is an enhancer of *Dm*Smn loss of function growth/survival defects. At least 100 animals of each genotype were scored in four replicates for the lethality assay. No significant variation was observed between control and experimental crosses with each independent trial. B) The *Drosophila* d02114 allele likely eliminates Fim function and in homozygous animals modestly perturbs NMJ morphology at larval muscle 4. Fim^d02114^ enhances *Dm*Smn loss of function NMJ defects consistent with studies in vertebrates [37]. All strains carry the ubiquitous TubGAL4 driver. Significance of p≤0.05 *versus* single mutant strains was determined by ANOVA and is indicated with asterisk; S.E.M. is shown. In representative images, red corresponds to anti-Discs Large (DLG), green is anti-synaptotagmin and blue is DAPI; scale bar indicates 15 microns.

First, we determined if Fimbrin (Fim), the *Drosophila* ortholog of PLS3, modifies *Dm*Smn loss of function defects in growth and NMJ assays. It has been shown that RNAi knockdown of *Dm*Smn (*Dm*Smn RNAi) results in 44% lethality in early pupal stages with 56% lethality at late pupal stages [26]. Loss of Fim alone does not cause larval or pupal lethality (data not shown). Three Fim loss of function alleles were crossed into the *Dm*Smn(RNAi) background and each accelerated death compared to *Dm*Smn(RNAi) control animals (Figure 5A).

In *Drosophila*, loss of SMN function results in a dose-dependant decrease in process arborization at the NMJ and diminished numbers of synaptic specializations, termed synaptic boutons [26]. Boutons are visualized as coincident pre-synaptic synaptotagmin and post-synaptic Discs large protein immunoreactivity. The number of synaptic boutons found between *Drosophila* neurons and muscles provides a simple and readily quantifiable assessment of phenotypic severity. We determined if the *Drosophila* PLS3 ortholog Fim might also modify the NMJ defects of *Dm*Smn. RNAi knockdown of *Dm*Smn using the ubiquitous tubulin promoter (TubGAL4;SmnRNAi) modestly decreased synaptic innervation in *Drosophila* larvae (reported as bouton numbers per muscle area, Figure 5B). Loss of *Drosophila* Fim function in Fim^d02114^ animals also modestly decreased bouton density. We found that effects of Fim^d02114^ and *Dm*Smn knockdown were synergistic; bouton numbers were significantly decreased suggesting that loss of Fim function exacerbated *Dm*Smn loss of function defects, being consistent with studies in vertebrate models of SMA [37]. These results suggest that PLS3 is a cross-species modifier of SMN function.

Next, *Drosophila* orthologs of candidate SMN modifier genes from *C. elegans* were examined. Cbp20 and Fmrf were selected as *Drosophila* orthologs of *ncbp-2* and *flp-4*, respectively, based on similarity and *Drosophila* loss of function alleles were obtained. (There are 32 genes in *C. elegans* encoding 32 FMRFamide-related neuropeptides, in contrast, three FMRFamide genes exist in *Drosophila*. There may be less redundancy in FMRFamide gene function in *Drosophila* [68], [69], [70]).

**Figure 6:**
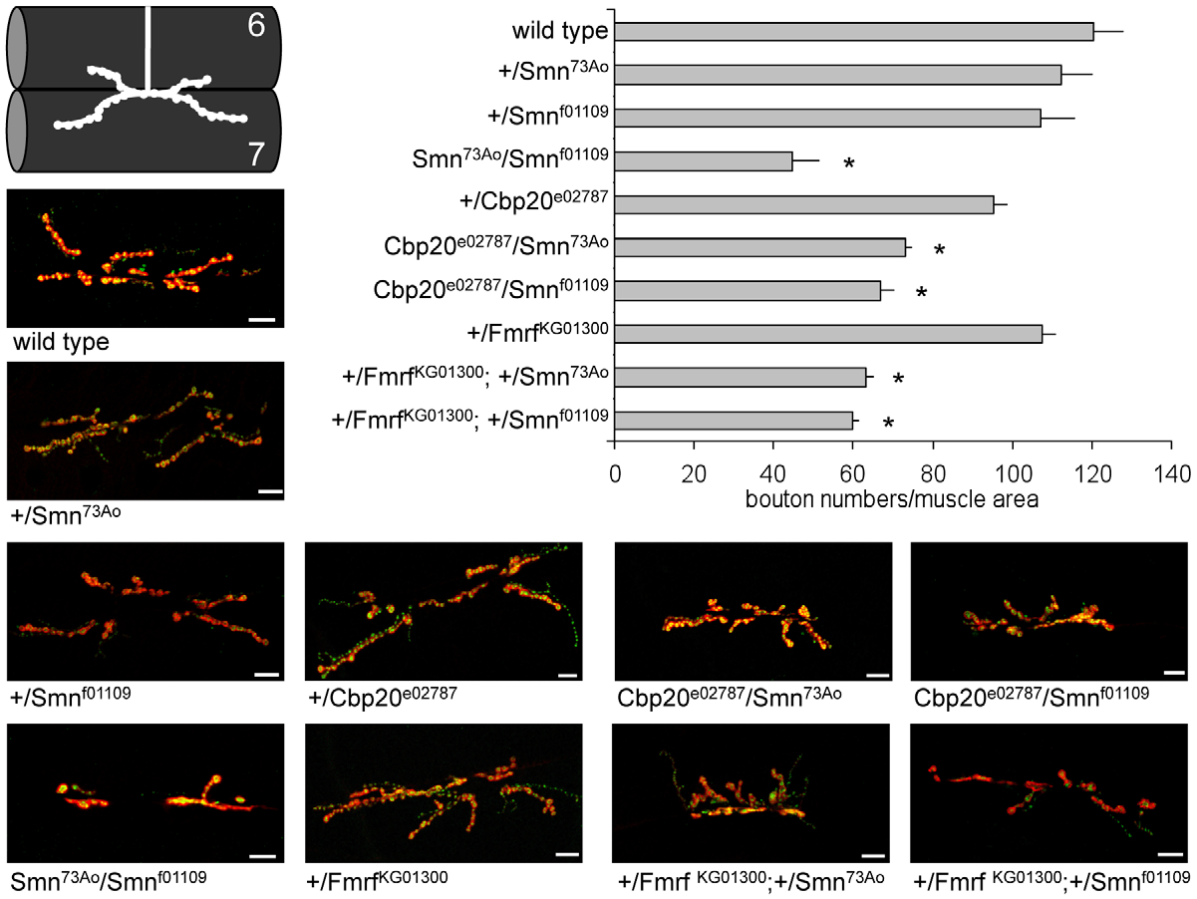
Loss of *Drosophila* Cbp20 or FMRF enhances *Dm*Smn loss of function neuromuscular defects. For two *C. elegans* modifier genes, loss of function alleles were available for orthologous *Drosophila* genes: Cbp20 and FMRF [68]–[70]. Loss of either *Drosophila* gene enhanced the NMJ defects of *Dm*Smn animals. The number of synaptic boutons in the A2 segment of muscles 6 and 7 was counted in third instar larvae (visualized using Discs large and synaptotagmin immunoreactivity, shown in red and green, respectively). Homozygous loss of *Dm*Smn in *Dm*Smn^f1109^/*Dm*Smn^73Ao^ animals dramatically decreases bouton number but loss of one copy of *Dm*Smn, Cbp20 or FMRF had little effect. Loss of one copy of either Cbp20 or FMRF in animals heterozygous for either *Dm*Smn allele significantly decreased bouton numbers. This nonallelic noncomplementation suggests a strong genetic interaction between these two conserved modifier genes and *Dm*Smn. Scale bar indicates 10 microns in representative images; S.E.M. is shown in graph; all transheterozygous combinations are significantly different from the corresponding single heterozygous controls with p≤0.05 by ANOVA as indicated with asterisk.

Heterozygous loss of *Dm*Smn function in +/Smn^73Ao^ or +/Smn^f01109^ animals had no significant effect on bouton number as expected, Smn^73Ao^/Smn^f01109^ animals had dramatically decreased bouton numbers (Figure 6). Loss of one copy of Cbc20 or Fmrf modestly decreased synaptic bouton number compared to control animals. However, simultaneous loss of one copy of *Dm*Smn and one copy of either modifier gene resulted in further synaptic bouton loss (Figure 6). The genetic interaction in trans-heterozygous animals is consistent with a strong genetic interaction between Smn and the two modifier genes. We were unable to obtain classical alleles of the *grk-2 Drosophila* ortholog. We conclude that Cbp20 and FMRFamide are conserved invertebrate enhancers of Smn loss of function defects and that this genetic interaction is conserved across species.

### Testing *Drosophila* modifier genes in *C. elegans* assays

A previous *Drosophila* screen identified twenty-seven P-element insertion lines that altered *Drosophila* SMN (*DmSmn*) loss of function defects [26]. Cross-species validation of these genes might also help elucidate conserved pathways that are critical in SMN loss of function pathology. However, several genes flanked the P-element insertion site for many of these modifier lines and the precise *DmSmn* modifier gene could not be unambiguously identified. Therefore, 40 candidate modifier genes were reported [26]. We identified the likely *C. elegans* orthologs for 32 of these 40 genes using reciprocal BLAST similarity searching (Table S1). The ability of these genes to modify *Cesmn-1(lf)* growth defects was assessed by feeding *Cesmn-1(lf)* and *+/Cesmn-1(lf)* animals bacteria expressing the corresponding dsRNA; RNAi feeding clones were constructed for *B0432.13*, *dhs-22* and *ugt-49* [53]. Twelve genes crossed species and modified *Cesmn-1(lf)* defects in one or both *C. elegans* assays.

### Orthologs of ten *Drosophila* genes modified *Cesmn-1(lf)* growth defects

Knockdown of seven *C. elegans* genes (*uso-1*, *nhr-85*, *egl-15*, *atf-6*, *ape-1*, *kcnl-2 and nekl-3*) orthologous to *Dm*Smn modifier genes specifically enhanced *Cesmn-1(lf)* growth defects, but did not significantly alter the percentage of large heterozygous *+/Cesmn-1(lf)* animals. Knockdown of the *C. elegans* ortholog *atn-1* significantly suppressed the growth defects of *Cesmn-1(lf)* animals without altering the percentage of large *+/Cesmn-1(lf)* siblings. Finally, *C. elegans* orthologs of two *Drosophila* genes were identified, whose genetic interaction with *Cesmn-1* resembled the interaction of *plst-1* with *Cesmn-1*: *cash-1* and *dlc-1*. RNAi knockdown of these two genes increased the percentage of large animals in the *+/Cesmn-1(lf)* population without altering the *Cesmn-1(lf)* population. Growth assay results for these ten genes are found in Table 1 (Rows 4 through 13), results for all orthologs tested can be found in Table S1, and a discussion of modifier gene function is presented in Text S2.

For *bona fide* cross-species modifier genes, the impact of modifier genes on SMN loss of function defects should be conserved across species (*i.e.* enhancer genes should enhance in both species). For six cross-species genes, the impact of modifier gene loss on *Dm*Smn and *Cesmn-1* loss of function defects was conserved as expected. Specifically, the enhancement of *Cesmn-1(lf)* defects by RNAi knockdown of *nhr-85*, *egl-15*, and *kcnl-2* was consistent with the effects of the corresponding *Drosophila* modifier genes on *Dm*Smn [26]; the corresponding *Drosophila* insertion lines (d09801, f02864, and d03336) enhanced *Dm*Smn defects and the transposon insertion in these lines are predicted to decrease function. The results for *Drosophila* orthologs of *C. elegans uso-1* and *nekl-3* were also consistent across species. The exacerbation of *Cesmn-1(lf)* growth defects observed after *uso-1(RNAi)* or *nekl-3(RNAi)* knockdown was consistent with the suppression of *Dm*Smn defects observed after over-expression of the cognate *Drosophila* genes. There was also good concordance for the effect of actinin orthologs across invertebrate species. The d00712 *Drosophila* insertion line likely drives over-expression of the *Drosophila* gene Actinin (Actn) and enhances *DmSmn* defects [26], [71], [72], while suppression of *Cesmn-1(lf)* growth defects by RNAi knockdown of *C. elegans atn-1* was observed here.

For four genes, it is unclear if the results for *Drosophila* orthologs are concordant across species: *atf-6*, *ape-1*, *dlc-1* and *cash-1*. For *atf-6* and *ape-1*, the corresponding *Drosophila* transposons (d05057 and d05779) are inserted into the 1^st^ intron of one of the two transcripts predicted for the orthologous *Drosophila* genes; accordingly, these transposons may perturb gene function or may drive over-expression of the predicted 2^nd^ shorter transcript. For the genes with complex genetic interactions with *Cesmn-1* (*i.e. dlc-1* and *cash-1*), the function of *Drosophila* orthologs ctp and CKA are likely decreased by *Drosophila* insertion lines f02345 and f04448, which suppressed and enhanced *Dm*Smn defects, respectively [26]. Overall, six of ten genes that modified *Dm*Smn growth defects are clearly concordant with the *C. elegans* growth data, suggesting conserved roles as SMN loss of function modifiers.

### Orthologs of three *Drosophila* genes modified *Cesmn-1(lf)* neuromuscular defects

*C. elegans* orthologs of *Dm*Smn modifier genes identified in the previous *Drosophila* screen [26] were also rescreened using the pharyngeal pumping assay. We found that RNAi knockdown *daf-4* enhanced *Cesmn-1(lf)* pharyngeal pumping defects, while knockdown of *kncl-2* or *nhr-25* suppressed *Cesmn-1(lf)* pumping defects (Table 3, rows 3 through 5).

**Table 3:** RNAi knockdown of candidate genes alters *Cesmn-1(lf)* neuromuscular defects in the pharyngeal pumping assay.

		*Cesmn-1(lf)*	*+/Cesmn-1(lf)*	*Cesmn-1(lf)*	*Cesmn-1(lf)*
		% change *vs.* control RNAi		*empty(RNAi)*	*candidate(RNAi)*
*Ce* gene	*Dm* gene			Pumps/min	
*Cesmn-1*	Smn	56±19*	101±2	53±9	27±6
*plst-1*	Fim	86±21*	106±1	53±9	43±8
*daf-4*	Wit	71±27*	93±2	38±7	26±6
*kcnl-2*	SK	147±50*	90±1	42±7	48±8
*nhr-25*	Usp	155±45*	91±5	48±8	67±11

*C. elegans* orthologs of *Dm*Smn modifier genes whose RNAi knockdown altered *Cesmn-1(lf)* pharyngeal pumping defects are listed in column 1 and column 2, respectively. The percent (%) change in pumping rates on empty vector RNAi *versus* candidate gene RNAi was determined independently in four separate experiments and is reported with S.E.M. for both *Cesmn-1(lf)* homozygous mutant and *+/Cesmn-1(lf)* heterozygous control animals (columns 3 and 4). As pumping rates for each genotype/treatment varied day-to-day (due to food thickness, *etc.*), % change *versus* empty vector RNAi controls is reported and was used to determine significance. The overall pharyngeal pumping rates in columns 5 and 6 were calculated by pooling all *Cesmn-1(lf)* animals reared with the RNAi bacteria indicated (n>40 animals). Animals were allowed to hatch on bacterial cultures carrying empty RNAi vector (*empty*) or RNAi constructs targeting *C. elegans* genes; pumping rates were determined after 3 days at the early adult or late L4 larval stage as illustrated in Figure 2. Significance (indicated with an asterisk) was determined by a two-sample *t*-test/Mann-Whitney *U* test according to sample-specific parameters (p≤0.05). Two additional genes that modified growth CG33172/*cash-1* and CG18375/*ape-1* (Table S2), reached significance here in pair-wise comparisons with controls. However, their knockdown enhanced *Cesmn-1(lf)* pumping defects in some trials and suppressed in other trials resulting in no significant change in overall pumping rates. See Table S2 for results for all genes tested and Materials and Methods for details.

*daf-4* encodes one of the *C. elegans* TGF-beta receptor subunits orthologous to *Drosophila* Wit (Witless). In *C. elegans*, *daf-4* and TGF-beta/Dpp pathway function is required for cell specification at numerous stages and for transit through the stress-resistant, long-lived dauer stage [73]. RNAi knockdown of *daf-4* exacerbated *Cesmn-1(lf)* pumping defects, consistent with the effect of TGF-beta pathway manipulation in *Drosophila* [26].

RNAi knockdown of two *C. elegans* genes diminished *Cesmn-1(lf)* pumping defects: *kcnl-2* and *nhr-25*. *kcnl-2* encodes a likely *C. elegans* SK channel subunit and *nhr-25* is one of the two *C. elegans* proteins most similar to *Drosophila* Usp (Ultraspiracle). No clear ortholog of Usp is found in the *C. elegans* genome. The corresponding *Drosophila* d00712 transposon insertion line likely drives over-expression of Usp resulting in enhancement of *DmSmn* defects [26]. This is consistent with *C. elegans* results. By contrast, the impact of SK/*kcnl-2* loss in growth *versus* pumping assays is discordant. The d03336 transposon insertion is located in the SK gene, likely perturbs SK function, and enhances *Dm*Smn growth and *Drosophila* NMJ defects [26]. This is consistent with *kcnl-2(RNAi)* enhancement of *C. elegans* growth defects described above. The suppression of *Cesmn-1(lf)* pumping defects observed here after *kcnl-2* knockdown may reflect differences in the requirement for *kcnl-2* function in neuromuscular tissue and/or the relative inefficiency of RNAi knockdown in neurons.

### Modifier gene interactions implicate specific pathways critical in neurodegenerative disease

To address the specificity of the invertebrate SMN modifier genes, the impact of their RNAi knockdown was examined on an unrelated pharyngeal pumping defective strain. Loss of *egl-30 (ad805)*, which perturbs G_q_α function in *C. elegans*, decreases their pharyngeal pumping rates [74]. RNAi knockdown did not significantly alter *egl-30* pharyngeal pumping rates for any modifier gene (Table S3), suggesting that these genes are likely specific modifiers of SMN loss of function defects.

**Figure 7:**
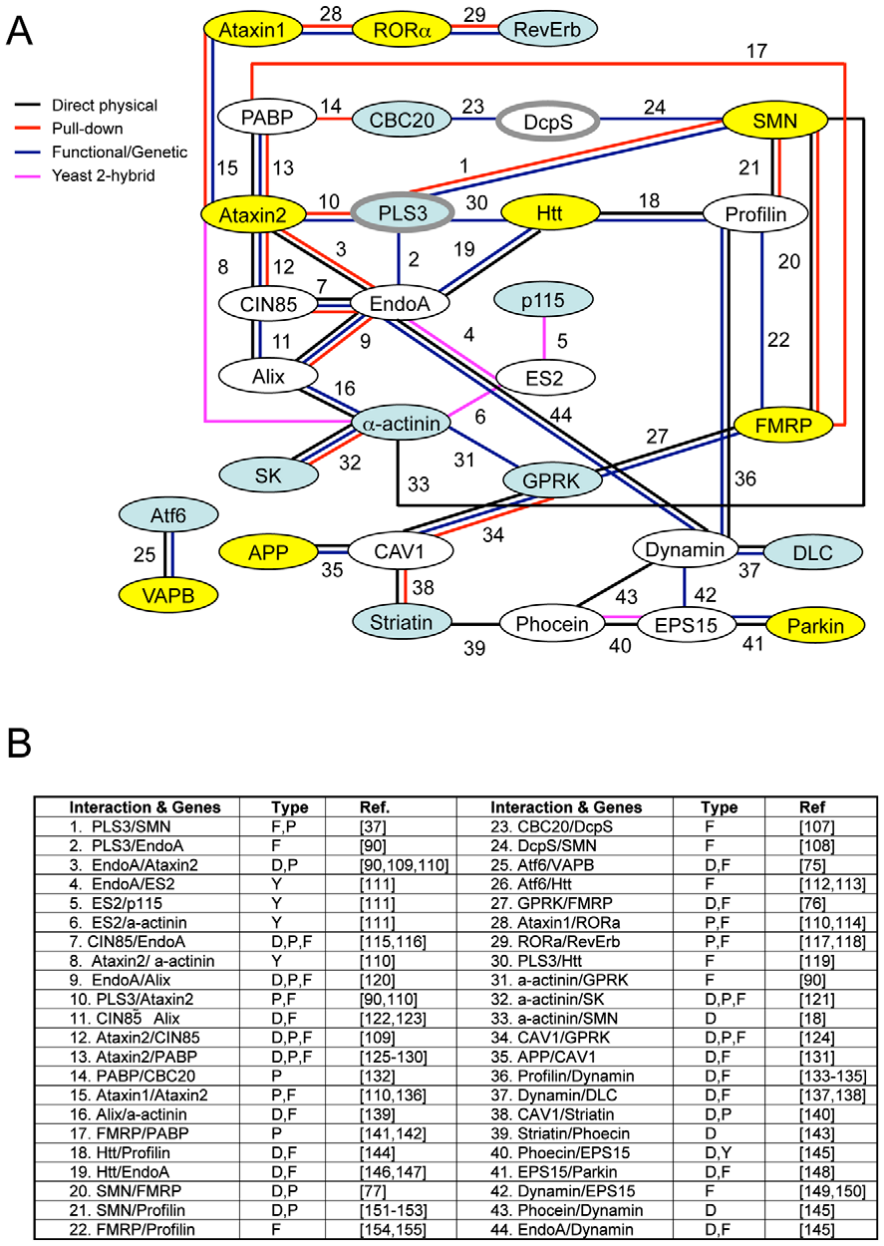
Interaction map of SMN modifier genes. A) Numerous published physical and/or functional interactions were found in the literature that connect many invertebrate SMN modifier genes. The names of vertebrate genes are used in the figure, but interactions are drawn from literature in any animal species. Each type of interaction is represented by different colors of connecting lines and references are provided in the table in part B. Not all interactions are indicated including interactions of FGF, TGF-beta and neuropeptide signaling pathways with endocytosis/cytoskeletal pathways, dynein interactions with APP [101] and possible connection between VAPB, the ALS8 locus, and SK channels through riluzole [102]–[106]. The interaction analysis here is not exhaustive and additional interactions may exist. B) The type of interaction is indicated in the table [107]–[155]: P pull-down, Y yeast 2-hybrid, F functional/genetic, D direct physical interaction. Genes shaded in blue are cross-species invertebrate modifiers; genes shaded in yellow are mammalian genes whose perturbation can cause neurodegeneration; genes outlined in grey are SMA modifiers in drug studies or in patients. Genes pertinent to endocytosis include CIN85, EndoA, Alix, PLS3, CAV1, GPRK, Ataxin2, Profilin, EPS15, Phocein, Dynamin, and Striatin; genes pertinent to RNA processing include PABP, CBC20, DcpS, SMN, and FMRP. Combined, our results and published studies support a model in which endocytosis and RNA translational control pathways are physically and functionally coupled [92], [93]. In normal animals, coupling of these seemingly disparate pathways may help coordinate synaptic activity and protein synthesis in the neuromuscular system.

Combined the results described here define eleven conserved genes that modify invertebrate SMN ortholog function in at least one assay in both *C. elegans* and *Drosophila* (summarized in Table 4). A subset of these cross-species modifier genes interact, directly or indirectly, with previously described neurological or neuromuscular disease proteins suggesting common neurodegenerative pathways may be at work (*i.e.* ATF6 with VAPB/ALS8 or GPRK2 and SMN1 with FMRP) [75]–[77]. To determine if specific cellular mechanisms could be implicated in SMN loss of function pathology, the published literature and public databases were examined for physical and/or functional interactions between cross-species SMN modifier genes, SMN and neuromuscular disease genes. A protein/genetic interaction map was assembled and is presented in Figure 7 with references. We note that genes implicated in endocytosis and mRNA translational regulation unexpectedly predominate in this interaction map. These two cellular pathways may be pertinent to SMN loss of function pathology.

**Table 4:** Summary: Invertebrate modifiers of SMN loss of function.

*Ce* gene	*Dm* gene	*Hs* gene	Change/Affect *Ce*	Change/Affect *Dm*
*plst-1*	Fim	Plastin 3 (PLS3)	RNAi/Cmpx Gr, Enh Pump	Lof/Enh
*daf-4*	Wit	TGFβ receptor (BMPRII)	RNAi/Enh Pump	Lof/Enh
*kcnl-2*	SK	SK channel (KCNN3)	RNAi/Enh Gr, Sup Pump≠	Lof/Enh
*nhr-25*	Usp	NHR LRH-1 (NR5A2)	RNAi/Sup Pump	OE/Enh
*uso-1*	p115	Vesicle docking (USO1)	RNAi/Enh Gr	OE/Sup
*nhr-85*	Eip75B	NHR RevErb (NR1D2)	RNAi/Enh Gr	Lof/Enh
*atf-6*	Atf6	Atf6 trans. factor (ATF6)	RNAi/Enh Gr	?/Sup
*egl-15*	Btl	FGF receptor (FGFR3)	RNAi/Enh Gr	Lof/Enh
*ape-1*	CG18375	p53 inhibition (PPP1R13)	RNAi/Enh Gr	?/Enh
*nekl-3*	Nek2	NIMA family kinase (NEK7)	RNAi/Enh Gr	OE/Sup
*atn-1*	Actinin	a-actinin (ACTN)	RNAi/Sup Gr	OE/Enh
*cash-1*	CG33172	Striatin (STRN)	RNAi/Cmpx Gr	Lof/Enh
*dlc-1*	cut up	Dynein light chain (DYNLL2)	RNAi/Cmpx Gr	Lof/Sup
*ncbp-2*	CBC20	Cap binding (CBP20)	RNAi/Enh Gr & Pump	Lof/Enh
*grk-2*	Gprk	GRK kinase (ADRBK1)	RNAi/Enh Gr & Pump	n.d.
*flp-4*	FMRF	Neuropeptide (NPFF)	RNAi/Enh Gr	Lof/Enh
*T02G5.3*	none	none	RNAi/Enh Gr, Sup Pump	n.d.

The summary table is divided into three sections corresponding to potential *Cesmn-1(lf)* modifier genes originally identified in humans, *Drosophila*, or *C. elegans* (1 in *Hs*, 40 in *Dm*, and 4 in *Ce*, respectively). Species and gene names are listed in the first three columns. The change in gene expression due to *Drosophila* GAL4-driven transposon insertion lines or *C. elegans* RNAi knockdown is listed in columns 4 and 5; effect on *Drosophila* gene function is predicted based solely on transposon insertion sites in some cases. (RNAi: RNAi knockdown; Lof: loss of function; decreased function or antisense; OE: over-expression; ?: unclear; Enh: enhanced SMN loss of function defects; Sup: suppressed SMN loss of function defects, Cmpx: complex genetic interaction; not determined, n.d.). Column 4 includes the *C. elegans* assay revealing modifier activity (Gr: growth; Pump: pharyngeal pumping.) RNAi knockdown was sufficient to modify pumping rates for *T02G5.3* and *ncbp-2*; mutant alleles of *grk-2* exacerbated *Cesmn-1(lf)* pumping defects. *flp-4* loss decreased *Cesmn-1(lf)* pumping rates in 4 out of 5 trials, but did not reach significance overall. Discordance between *Drosophila* and *C. elegans* results is indicated with ≠.

## Discussion

Enormous effort over the last few decades has resulted in the successful identification of numerous neurodegenerative disease genes and the proteins they encode. However, in many cases there remains considerable controversy as to why perturbation of these genes results in neurodegeneration [78]–[81]. SMN plays a well-described and ubiquitous role in the Gemin complex and snRNP assembly [8]–[10], yet SMA specifically affects neuromuscular function, motorneuron survival, and leads to muscle atrophy. Given this neuromuscular specificity, it seems likely that loss of SMN function impacts cellular pathways outside of the Gemin complex. In addition, given the complexity of cellular signaling pathways, genetic pathways that are not directly involved in SMN activity may impact SMN loss of function pathology. To identify SMN modifier pathways, we have used a genetic approach. Unbiased genetic screens are powerful tools as they utilize functional criteria for the identification of genes critical for cellular function. In the case of SMN loss of function, genetic screens can reveal conserved genes and pathways that are important for neuromuscular dysfunction and pathology independent of initial assumptions about the roles of SMN in neurons and muscles. The identification of hitherto unsuspected molecular pathways that modulate SMN neuropathology, directly or indirectly, is expected to widen the range of targets for SMA therapy development.

Conserved genes that modify SMN loss of function defects in disparate species likely represent pathways that are important for SMN loss of function defects or pathology. *C. elegans* and *Drosophila* models have been used here to identify SMN loss of function modifier genes that ‘cross species’. It is difficult to estimate how many modifiers of SMN loss of function were missed in the genome-wide *C. elegans* RNAi screen. ‘Growth’ encompasses a variety of factors; slow progression through the larvae stages, reduced growth in the adult stage, longevity, body size, different culture format (liquid *versus* plates), or a combination of these. Additionally, identification of genetic modifiers for a null allele can be more challenging as compared to identification of genetic modifiers for partial loss of function alleles [82]. No genetic screen can identify all modifier genes pertinent to a pathway and important players can be missed (*e.g.* miRNAs). Despite this, there is excellent concordance of modifier gene action in *C. elegans* and *Drosophila*. In most cases, genes that enhanced SMN loss of function defects in *Drosophila* also enhanced SMN loss of function defects in *C. elegans* and *vice versa*. This concordance suggests that the genetic relationships between SMN and these modifier genes are conserved across species. Orthologous genes are likely also important in SMN loss of function pathology in vertebrate species, as suggested by other invertebrate modifier screens that have identified conserved human disease-related genes and/or functional pathways [83]–[88].

Thus far, there are only two published human SMA modifier genes: Plastin 3 (PLS3) and SMN2. The role of SMN2 is clear as it provides a modicum of functional SMN protein. However, the role of PLS3 in SMA is controversial and it is unclear how PLS3 levels might modulate severity in SMA patients [37], [43]. We find that invertebrate PLS3 orthologs act as modifiers in *C. elegans* and *Drosophila* models. This cross-species interaction of PLS3 and SMN both increases confidence in the invertebrate models and suggests that plastin-associated pathways are important for SMN function at a fundamental level in multiple contexts.

In the bioinformatic analysis presented in Figure 7, we independently identified two cellular pathways that connect multiple modifier genes with SMN: endocytosis and RNA processing/translational control pathways. Regarding the former, it is of note that the yeast ortholog of PLS3, Sac6p, is a key player in endocytosis and Sac6p levels are critical when expanded polyglutamine neurodegenerative disease proteins are expressed in this system [42], [89]–[91]. We suggest that 1) these two cellular mechanisms may be of particular importance in SMA pathology and 2) that unexpected and intimate connections exist between these two pathways. A pair of recently published studies found that the microRNA regulatory RISC complex and endocytosis are physically and functionally coupled in non-neuronal cells [92], [93]. Interestingly, the RISC complex also contains Gemin complex proteins; the function of the Gemin and RISC complexes may be related, directly or indirectly [20]. We speculate that in normal animals, physically coupling the seemingly disparate pathways of endocytosis and local translational regulation may help coordinate synaptic activity and receptor signaling with protein translation during both synaptic development and neuron maintenance [94]. Defects in endocytosis have been suggested previously to play a pivotal role in neurodegenerative diseases in numerous scenarios. In such diseases, including SMA, perturbation of endocytosis may result in RNA translational control defects, or *vice versa* [92], [93]. A recent study has demonstrated impaired synaptic vesicle release at the NMJs in severe SMA mice consistent with defects in synaptic vesicle endocytosis/recycling and/or defects in active zone organization [94]. Further studies are warranted to ascertain the interdependence of endocytosis with translational control pathways and to explore the relevance of these pathways in neurodegenerative disease.

## Materials and Methods

### Genetic analysis

*Cesmn-1(lf)* homozygous strains cannot be maintained due to infertility; *hT2(lethal)[myo-2p::GFP]/Cesmn-1(lf)* (*hT2[bli-4(e937) let-?(q782)qIs48] (I;III)*) animals are fertile and were maintained using standard techniques [95]. The lack of homologous pairing for the rearranged chromosomes LGI and LGIII in *hT2* animals likely results in increased maternal/zygotic expression of *Cesmn-1* and other balanced genes [96]. As expected, we found that the progeny of *hT2* animals were relatively resistant to *Cesmn-1(RNAi)* compared to wild type control strains in our assays (data not shown). Consequently, to keep the genetic background invariant, all animals were tested herein were the progeny of *hT2(lethal)[myo-2p::GFP]* parents. The use of RNAi sensitive *C. elegans* mutant strains was avoided as their behavior is not normal in many assays (Hart, unpublished observations) and because SMN complex/Sm proteins have been implicated in miRNA pathways [20], [97], [98]. We note that RNAi knockdown is not always effective. To control for genetic background effects, animals tested in these studies were either heterozygous for *hT2* balancer chromosome or progeny of *hT2* parents unless otherwise noted.

*plst-1(tm4255)* animals were obtained from the Japanese National Bioresource Project and were backcrossed four times before further study. The *tm4255* allele is a 368 base pair deletion that removes one of the calponin-like, actin-binding homology (CH) domains; *plst-1(tm4255)* is likely a partial loss of function allele. To test the genetic interaction of *plst-1* with *Cesmn-1*, the backcrossed *plst-1(tm4255)* allele was used to create a double mutant with *Cesmn-1(lf)*. *The flp-4(yn35)* deletion allele was isolated by PCR-based screening of EMS-mutagenized animals. The *yn35* allele is a 928 base pair deletion that removes exon 3 of *flp-4* gene along with 5′ sequences (flanking sequences, ttctgaaaaacttttaataa and agctcgccgagccgagtctt) [66]. The *grk-2(rt97)* loss of function allele was previously characterized [60].

*Drosophila* stocks were maintained on standard cornmeal/yeast/molasses/agar medium at 25°C. The mutations of *Smn* ^73Ao^ and *Smn* ^f01109^ have been described previously [25]. *Cbp20* ^e02787^ is a Piggy-Bac insertion mutation from the Exelixis collection. The insertion location is 5′ upstream and adjacent to the start codon of the Cbp20 transcript. *Fmrf* ^KG1300^ and Fim alleles are loss of function alleles (Flybase). The line d03334 may have an unlinked lethal mutation on another chromosome. Fim^d02114^ and SmnRNAi; Fim^d02114^ have Tubulin:Gal4 in the background; this Gal4 transgene does not alter Smn defects (data not shown).

### Bioinformatics

*C. elegans* orthologs of *Drosophila* and human genes were identified by BLAST searching at NCBI. When a clear ortholog was not identified by reciprocal BLAST analysis, the most similar *C. elegans* genes were generally tested. *plst-1* corresponds to exons of predicted adjacent genes Y104H12BR.1 and Y104H12BL.1 based on similarity searching. *T02G5.3* corresponds to exons of *T02G5.3*, *T02G5.2*, and *T02G5.1* based on high-throughput cDNA sequencing and gene prediction programs [99]. New gene predictions have been reported to Wormbase. To assemble the interaction map in Figure 7, literature pertaining to each modifier gene was examined at NCBI, AceView, *C. elegans* and yeast on-line databases (Wormbase and SGD) to identify functional or direct interactions between modifier genes and neurodegenerative disease genes.

### Construction of RNAi feeding clones for *B0432.13*, *dhs-22* and *ugt-49*

The L4440 vector [47] was used to clone PCR products corresponding to *B0432.13*, *dhs-22* and *ugt-49* genes. Plasmids were transformed into the bacterial strain HT115(DE3) [47], [49]. Primers used for cloning were: *B0432.13* forward 5′-acaagctctcgacatcgctg-3′, reverse 5′- ttaatcgccgcatcctcttg -3′; *dhs-22* forward 5′-tatgctgtgcagaagcgaag-3′, reverse 5′-ctgcttgattcctggtgtattc-3′; *ugt-49* forward 5′-acgtggatgtagctgaatgg-3′, reverse 5′- acgtgaagaacagcaacgaac-3′.

### *C. elegans* growth studies

For analysis of modifier genes, animals were reared for two generations/5 days on plates spread with bacterial RNAi strains from the Ahringer or Vidal RNAi libraries [53]. RNAi clones corresponding to modifier genes in Table 4 were sequenced to confirm accuracy. The *hlh-4(RNAi)* clone in the feeding library was incorrect. A *Cesmn-1(ok355)*;*hlh-4(tm604)* double mutant strain was generated. *hlh-4(tm604)* did not affect the pharyngeal pumping rates of *Cesmn-(lf)* (data not shown) and *hlh-4* was excluded from further analysis. Length and GFP fluorescence was determined using the COPAS Biosorter (Union Biometrica, Holliston, MA) and the percentage of large animals was determined for each genotype [51]. Three to six independent determinations were undertaken for each genotype/RNAi culture. Significant changes from *empty(RNAi)* were calculated for each RNAi/genotype using the two-tailed Mann-Whitney *U* test.

### *C. elegans* pharyngeal pumping

The average pharyngeal pumping rates of animals were determined after 3 days (at 25, 25 and 20°C) post-hatching on empty vector (*empty(RNAi)*) or candidate gene RNAi bacterial feeding strains. Animals were videotaped while feeding for 10 seconds with an AxioCam ICc1 camera on a Zeiss Stemi SV11 at 20 to 66× magnification. Movies were slowed before counting pumping rates. Pharyngeal grinder movements in any axis were scored as a pumping event. Average pumping rates (± standard error of the mean, S.E.M) for each genotype/treatment were calculated independently in two to four separate experiments. The percent change in pumping rate on empty vector *versus* candidate gene RNAi was determined for each trial for both *Cesmn-1(lf)* homozygous and *+/Cesmn-1(lf)* heterozygous animals and used to calculate the mean, S.E.M, and significance.

### *C. elegans* genome-wide RNAi screen

*hT2(bli-4(e937) let-?(q782) qIs48[myo-2p::GFP]) (I;III)* animals were reared in liquid cultures in a 96-well plate format on RNAi feeding strains [53]. At least two independent cultures corresponding to each *C. elegans* RNAi feeding clone were established. Concentrated dsRNA expressing bacteria was added to cultures as necessary to prevent starvation. Cultures were maintained for 8 days at 25°C to generate sufficient animals for analysis. Length and fluorescence were determined using the COPAS BioSorter (Union Biometrica, Holliston, MA). Data was exported to Excel (Microsoft Corp.) for analysis. Thirty-one clones were identified that modified the average length of *Cesmn-1(lf)* animals relative to *+/Cesmn-1(lf)* siblings in both trials. Four of these genes altered *Cesmn-1(lf)* size relative to *+/Cesmn-1(lf)* siblings in at least 40% of subsequent trials and these were selected as candidate modifier genes for neuromuscular analysis as described in the text.

### *Drosophila* pupal lethality

Three males and three virgin females were placed on fresh food at 25°C on day 1. Eggs were collected for next 2 days (Set 1), and the parents transferred to fresh food. Eggs were collected for another 2 days (Set 2), and the parents discarded. The F1 animals were scored after 15 days,- on the 16^th^ day for the first set, and the 19th day for the second set, from day 1. White pupae were scored as early stage death and black pupae were scored as late stage death. Control crosses of tubGAL4:FL26B(Smn RNAi) out-crossed to the wild type strain were used as a control for every experimental set. Significance was determined by Chi-square analysis.

### *Drosophila* NMJ analysis

Primary antibodies were used at the following dilutions: monoclonal anti-DLG (1∶500) (Developmental Studies Hybridoma Bank), polyclonal anti-Synaptotagmin (1∶1000) (a gift from Hugo Bellen). FITC- (1∶40) and Cy5- (1∶40) conjugated anti-rabbit and anti-mouse secondary antibodies were purchased from Jackson Immunoresearch Laboratories. Anti-Disc large used at 1∶100 (Hybridoma) and anti-HRP used at 1∶1000 (Cappell). 3^rd^ instar larvae were dissected and fixed for 5 minutes in Bouin's fixative. Stained specimens were mounted in FluoroGuard Antifade Reagent (Bio-Rad), and images were obtained with a Zeiss LSM510 confocal microscope. Bouton numbers were counted based on the Discs large and Synaptotagmin staining in the A2 segment between muscles 6 and 7 or muscle 4 as indicated. The ratio of muscle area for the various genotypes was normalized to wild type. At least 10–12 animals of each genotype were dissected for the bouton analysis. The ANOVA multiple comparison test was used for statistical analysis.

## Supporting Information

Table S1 Summary of *C. elegans* growth assays.
(0.09 MB DOC)

Table S2 Summary of *C. elegans* pharyngeal pumping assays.
(0.09 MB DOC)

Table S3 Invertebrate modifier genes specifically affect *Cesmn-1(lf)* pharyngeal pumping defects.
(0.05 MB DOC)

Table S4 Body length determinations for *Cesmn-1(lf)* modifier genes.
(0.05 MB DOC)

Text S1 Methods for supplementary files.
(0.02 MB DOC)

Text S2 Supplementary discussion of invertebrate modifier genes.
(0.33 MB DOC)

## References

[1] Lefebvre S, Burglen L, Reboullet S, Clermont O, Burlet P (1995). Identification and characterization of a spinal muscular atrophy-determining gene.. Cell.

[2] Boda B, Mas C, Giudicelli C, Nepote V, Guimiot F (2004). Survival motor neuron *SMN1* and *SMN2* gene promoters: identical sequences and differential expression in neurons and non-neuronal cells.. Eur J Hum Genet.

[3] Monani UR, McPherson JD, Burghes AH (1999). Promoter analysis of the human centromeric and telomeric survival motor neuron genes (SMNC and SMNT).. Biochim Biophys Acta.

[4] Pearn J (1980). Classification of spinal muscular atrophies.. Lancet.

[5] Cusin V, Clermont O, Gerard B, Chantereau D, Elion J (2003). Prevalence of *SMN1* deletion and duplication in carrier and normal populations: implication for genetic counselling.. J Med Genet.

[6] McAndrew PE, Parsons DW, Simard LR, Rochette C, Ray PN (1997). Identification of proximal spinal muscular atrophy carriers and patients by analysis of SMNT and SMNC gene copy number.. Am J Hum Genet.

[7] Ogino S, Leonard DG, Rennert H, Ewens WJ, Wilson RB (2002). Genetic risk assessment in carrier testing for spinal muscular atrophy.. Am J Med Genet.

[8] Gubitz AK, Feng W, Dreyfuss G (2004). The SMN complex.. Exp Cell Res.

[9] Ohn T, Kedersha N, Hickman T, Tisdale S, Anderson P (2008). A functional RNAi screen links O-GlcNAc modification of ribosomal proteins to stress granule and processing body assembly.. Nat Cell Biol.

[10] Hua Y, Zhou J (2004). Survival motor neuron protein facilitates assembly of stress granules.. FEBS Lett.

[11] Fan L, Simard LR (2002). Survival motor neuron (SMN) protein: role in neurite outgrowth and neuromuscular maturation during neuronal differentiation and development.. Hum Mol Genet.

[12] Rossoll W, Jablonka S, Andreassi C, Kroning AK, Karle K (2003). Smn, the spinal muscular atrophy-determining gene product, modulates axon growth and localization of beta-actin mRNA in growth cones of motoneurons.. J Cell Biol.

[13] Francis JW, Sandrock AW, Bhide PG, Vonsattel JP, Brown RH (1998). Heterogeneity of subcellular localization and electrophoretic mobility of survival motor neuron (SMN) protein in mammalian neural cells and tissues.. Proc Natl Acad Sci U S A.

[14] Giavazzi A, Setola V, Simonati A, Battaglia G (2006). Neuronal-specific roles of the survival motor neuron protein: evidence from survival motor neuron expression patterns in the developing human central nervous system.. J Neuropathol Exp Neurol.

[15] Pagliardini S, Giavazzi A, Setola V, Lizier C, Di Luca M (2000). Subcellular localization and axonal transport of the survival motor neuron (SMN) protein in the developing rat spinal cord.. Hum Mol Genet.

[16] Gennarelli M, Lucarelli M, Capon F, Pizzuti A, Merlini L (1995). Survival motor neuron gene transcript analysis in muscles from spinal muscular atrophy patients.. Biochem Biophys Res Commun.

[17] Coovert DD, Le TT, McAndrew PE, Strasswimmer J, Crawford TO (1997). The survival motor neuron protein in spinal muscular atrophy.. Hum Mol Genet.

[18] Rajendra TK, Gonsalvez GB, Walker MP, Shpargel KB, Salz HK (2007). A *Drosophila melanogaster* model of spinal muscular atrophy reveals a function for SMN in striated muscle.. J Cell Biol.

[19] Walker MP, Rajendra TK, Saieva L, Fuentes JL, Pellizzoni L (2008). The SMN complex localizes to the sarcomeric Z-disc and is a proteolytic target of calpain.. Hum Mol Genet.

[20] Mourelatos Z, Dostie J, Paushkin S, Sharma A, Charroux B (2002). miRNPs: a novel class of ribonucleoproteins containing numerous microRNAs.. Genes Dev.

[21] Pellizzoni L, Baccon J, Charroux B, Dreyfuss G (2001). The survival of motor neurons (SMN) protein interacts with the snoRNP proteins fibrillarin and GAR1.. Curr Biol.

[22] Whitehead SE, Jones KW, Zhang X, Cheng X, Terns RM (2002). Determinants of the interaction of the spinal muscular atrophy disease protein SMN with the dimethylarginine-modified box H/ACA small nucleolar ribonucleoprotein GAR1.. J Biol Chem.

[23] Cifuentes-Diaz C, Frugier T, Tiziano FD, Lacene E, Roblot N (2001). Deletion of murine SMN exon 7 directed to skeletal muscle leads to severe muscular dystrophy.. J Cell Biol.

[24] Shafey D, Cote PD, Kothary R (2005). Hypomorphic Smn knockdown C2C12 myoblasts reveal intrinsic defects in myoblast fusion and myotube morphology.. Exp Cell Res.

[25] Chan YB, Miguel-Aliaga I, Franks C, Thomas N, Trulzsch B (2003). Neuromuscular defects in a *Drosophila* survival motor neuron gene mutant.. Hum Mol Genet.

[26] Chang HC, Dimlich DN, Yokokura T, Mukherjee A, Kankel MW (2008). Modeling spinal muscular atrophy in *Drosophila*.. PLoS ONE.

[27] McWhorter ML, Monani UR, Burghes AH, Beattie CE (2003). Knockdown of the survival motor neuron (Smn) protein in zebrafish causes defects in motor axon outgrowth and pathfinding.. J Cell Biol.

[28] Kariya S, Park GH, Maeno-Hikichi Y, Leykekhman O, Lutz C (2008). Reduced SMN protein impairs maturation of the neuromuscular junctions in mouse models of spinal muscular atrophy.. Hum Mol Genet.

[29] Lorson CL, Androphy EJ (2000). An exonic enhancer is required for inclusion of an essential exon in the SMA-determining gene SMN.. Hum Mol Genet.

[30] Monani UR, Coovert DD, Burghes AH (2000). Animal models of spinal muscular atrophy.. Hum Mol Genet.

[31] Monani UR, Lorson CL, Parsons DW, Prior TW, Androphy EJ (1999). A single nucleotide difference that alters splicing patterns distinguishes the SMA gene *SMN1* from the copy gene *SMN2*.. Hum Mol Genet.

[32] Lefebvre S, Burlet P, Liu Q, Bertrandy S, Clermont O (1997). Correlation between severity and SMN protein level in spinal muscular atrophy.. Nat Genet.

[33] Feldkotter M, Schwarzer V, Wirth R, Wienker TF, Wirth B (2002). Quantitative analyses of *SMN1* and *SMN2* based on real-time lightCycler PCR: fast and highly reliable carrier testing and prediction of severity of spinal muscular atrophy.. Am J Hum Genet.

[34] Wirth B, Herz M, Wetter A, Moskau S, Hahnen E (1999). Quantitative analysis of survival motor neuron copies: identification of subtle *SMN1* mutations in patients with spinal muscular atrophy, genotype-phenotype correlation, and implications for genetic counseling.. Am J Hum Genet.

[35] Lefebvre S, Burglen L, Frezal J, Munnich A, Melki J (1998). The role of the SMN gene in proximal spinal muscular atrophy.. Hum Mol Genet.

[36] Burghes AH (1997). When is a deletion not a deletion? When it is converted.. Am J Hum Genet.

[37] Oprea GE, Krober S, McWhorter ML, Rossoll W, Muller S (2008). Plastin 3 is a protective modifier of autosomal recessive spinal muscular atrophy.. Science.

[38] Bretscher A (1981). Fimbrin is a cytoskeletal protein that crosslinks F-actin in vitro.. Proc Natl Acad Sci U S A.

[39] Bretscher A, Weber K (1980). Fimbrin, a new microfilament-associated protein present in microvilli and other cell surface structures.. J Cell Biol.

[40] Glenney JR, Kaulfus P, Matsudaira P, Weber K (1981). F-actin binding and bundling properties of fimbrin, a major cytoskeletal protein of microvillus core filaments.. J Biol Chem.

[41] Adams AE, Botstein D, Drubin DG (1989). A yeast actin-binding protein is encoded by SAC6, a gene found by suppression of an actin mutation.. Science.

[42] Kubler E, Riezman H (1993). Actin and fimbrin are required for the internalization step of endocytosis in yeast.. EMBO J.

[43] Bowerman M, Anderson CL, Beauvais A, Boyl PP, Witke W (2009). SMN, profilin IIa and plastin 3: a link between the deregulation of actin dynamics and SMA pathogenesis.. Mol Cell Neurosci.

[44] Briese M, Esmaeili B, Fraboulet S, Burt EC, Christodoulou S (2009). Deletion of *smn-1*, the *Caenorhabditis elegans* ortholog of the spinal muscular atrophy gene, results in locomotor dysfunction and reduced lifespan.. Hum Mol Genet.

[45] Miguel-Aliaga I, Culetto E, Walker DS, Baylis HA, Sattelle DB (1999). The *Caenorhabditis elegans* orthologue of the human gene responsible for spinal muscular atrophy is a maternal product critical for germline maturation and embryonic viability.. Hum Mol Genet.

[46] McKim KS, Peters K, Rose AM (1993). Two types of sites required for meiotic chromosome pairing in *Caenorhabditis elegans*.. Genetics.

[47] Timmons L, Fire A (1998). Specific interference by ingested dsRNA.. Nature.

[48] Kamath RS, Martinez-Campos M, Zipperlen P, Fraser AG, Ahringer J (2001). Effectiveness of specific RNA-mediated interference through ingested double-stranded RNA in *Caenorhabditis elegans*.. Genome Biol.

[49] Timmons L, Court DL, Fire A (2001). Ingestion of bacterially expressed dsRNAs can produce specific and potent genetic interference in *Caenorhabditis elegans*.. Gene.

[50] Rual JF, Ceron J, Koreth J, Hao T, Nicot AS (2004). Toward improving *Caenorhabditis elegans* phenome mapping with an ORFeome-based RNAi library.. Genome Res.

[51] Pulak R (2006). Techniques for analysis, sorting, and dispensing of *C. elegans* on the COPAS flow-sorting system.. Methods Mol Biol.

[52] Huang LS, Sternberg PW (2006). Genetic dissection of developmental pathways.. WormBook.

[53] Kamath RS, Ahringer J (2003). Genome-wide RNAi screening in *Caenorhabditis elegans*.. Methods.

[54] Lall S, Piano F, Davis RE (2005). *Caenorhabditis elegans* decapping proteins: localization and functional analysis of Dcp1, Dcp2, and DcpS during embryogenesis.. Mol Biol Cell.

[55] Avery L (1993). The genetics of feeding in *Caenorhabditis elegans*.. Genetics.

[56] Sulston JE, Horvitz HR (1977). Post-embryonic cell lineages of the nematode, *Caenorhabditis elegans*.. Dev Biol.

[57] Sulston JE, Schierenberg E, White JG, Thomson JN (1983). The embryonic cell lineage of the nematode *Caenorhabditis elegans*.. Dev Biol.

[58] Simmer F, Moorman C, van der Linden AM, Kuijk E, van den Berghe PV (2003). Genome-wide RNAi of *C. elegans* using the hypersensitive *rrf-3* strain reveals novel gene functions.. PLoS Biol.

[59] Kennedy S, Wang D, Ruvkun G (2004). A conserved siRNA-degrading RNase negatively regulates RNA interference in *C. elegans*.. Nature.

[60] Fukuto HS, Ferkey DM, Apicella AJ, Lans H, Sharmeen T (2004). G protein-coupled receptor kinase function is essential for chemosensation in *C. elegans*.. Neuron.

[61] Barrett PL, Fleming JT, Gobel V (2004). Targeted gene alteration in *Caenorhabditis elegans* by gene conversion.. Nat Genet.

[62] Gengyo-Ando K, Mitani S (2000). Characterization of mutations induced by ethyl methanesulfonate, UV, and trimethylpsoralen in the nematode *Caenorhabditis elegans*.. Biochem Biophys Res Commun.

[63] Gilchrist EJ, O'Neil NJ, Rose AM, Zetka MC, Haughn GW (2006). TILLING is an effective reverse genetics technique for *Caenorhabditis elegans*.. BMC Genomics.

[64] Jansen G, Hazendonk E, Thijssen KL, Plasterk RH (1997). Reverse genetics by chemical mutagenesis in *Caenorhabditis elegans*.. Nat Genet.

[65] Lesa GM (2006). Isolation of *Caenorhabditis elegans* gene knockouts by PCR screening of chemically mutagenized libraries.. Nat Protoc.

[66] Liu T, Kim K, Li C, Barr MM (2007). FMRFamide-like neuropeptides and mechanosensory touch receptor neurons regulate male sexual turning behavior in *Caenorhabditis elegans*.. J Neurosci.

[67] Li C, Kim K (2008). Neuropeptides.. WormBook.

[68] Mount SM, Salz HK (2000). Pre-messenger RNA processing factors in the *Drosophila* genome.. J Cell Biol.

[69] Lasko P (2000). The *Drosophila melanogaster* genome: translation factors and RNA binding proteins.. J Cell Biol.

[70] Nambu JR, Murphy-Erdosh C, Andrews PC, Feistner GJ, Scheller RH (1988). Isolation and characterization of a *Drosophila* neuropeptide gene.. Neuron.

[71] Roulier EM, Fyrberg C, Fyrberg E (1992). Perturbations of *Drosophila* alpha-actinin cause muscle paralysis, weakness, and atrophy but do not confer obvious nonmuscle phenotypes.. J Cell Biol.

[72] Fyrberg E, Kelly M, Ball E, Fyrberg C, Reedy MC (1990). Molecular genetics of *Drosophila* alpha-actinin: mutant alleles disrupt Z disc integrity and muscle insertions.. J Cell Biol.

[73] Inoue T, Thomas JH (2000). Targets of TGF-beta signaling in *Caenorhabditis elegans* dauer formation.. Dev Biol.

[74] Brundage L, Avery L, Katz A, Kim UJ, Mendel JE (1996). Mutations in a *C. elegans* Gqalpha gene disrupt movement, egg laying, and viability.. Neuron.

[75] Gkogkas C, Middleton S, Kremer AM, Wardrope C, Hannah M (2008). VAPB interacts with and modulates the activity of ATF6.. Hum Mol Genet.

[76] Wang H, Wu LJ, Kim SS, Lee FJ, Gong B (2008). FMRP acts as a key messenger for dopamine modulation in the forebrain.. Neuron.

[77] Piazzon N, Rage F, Schlotter F, Moine H, Branlant C (2008). *In vitro* and *in cellulo* evidences for association of the survival of motor neuron complex with the fragile X mental retardation protein.. J Biol Chem.

[78] Bauer PO, Nukina N (2009). The pathogenic mechanisms of polyglutamine diseases and current therapeutic strategies.. J Neurochem.

[79] Crawford TO, Pardo CA (1996). The neurobiology of childhood spinal muscular atrophy.. Neurobiol Dis.

[80] Goedert M, Spillantini MG (2006). A century of Alzheimer's disease.. Science.

[81] Lang AE, Lozano AM (1998). Parkinson's disease. First of two parts.. N Engl J Med.

[82] Jorgensen EM, Mango SE (2002). The art and design of genetic screens: *Caenorhabditis elegans*.. Nat Rev Genet.

[83] Dimitriadi M, Hart AC (2010). Neurodegenerative disorders: Insights from the nematode *Caenorhabditis elegans*.. Neurobiol Dis.

[84] Fiuza UM, Arias AM (2007). Cell and molecular biology of Notch.. J Endocrinol.

[85] Kim SK (2007). Common aging pathways in worms, flies, mice and humans.. J Exp Biol.

[86] Leicht DT, Balan V, Kaplun A, Singh-Gupta V, Kaplun L (2007). Raf kinases: function, regulation and role in human cancer.. Biochim Biophys Acta.

[87] Schlegel A, Stainier DY (2007). Lessons from “lower” organisms: what worms, flies, and zebrafish can teach us about human energy metabolism.. PLoS Genet.

[88] Silverman GA, Luke CJ, Bhatia SR, Long OS, Vetica AC (2009). Modeling molecular and cellular aspects of human disease using the nematode *Caenorhabditis elegans*.. Pediatr Res.

[89] Kaksonen M, Toret CP, Drubin DG (2005). A modular design for the clathrin- and actin-mediated endocytosis machinery.. Cell.

[90] Ralser M, Nonhoff U, Albrecht M, Lengauer T, Wanker EE (2005). Ataxin-2 and huntingtin interact with endophilin-A complexes to function in plastin-associated pathways.. Hum Mol Genet.

[91] Singer-Kruger B, Nemoto Y, Daniell L, Ferro-Novick S, De Camilli P (1998). Synaptojanin family members are implicated in endocytic membrane traffic in yeast.. J Cell Sci.

[92] Gibbings DJ, Ciaudo C, Erhardt M, Voinnet O (2009). Multivesicular bodies associate with components of miRNA effector complexes and modulate miRNA activity.. Nat Cell Biol.

[93] Lee YS, Pressman S, Andress AP, Kim K, White JL (2009). Silencing by small RNAs is linked to endosomal trafficking.. Nat Cell Biol.

[94] Kong L, Wang X, Choe DW, Polley M, Burnett BG (2009). Impaired synaptic vesicle release and immaturity of neuromuscular junctions in spinal muscular atrophy mice.. J Neurosci.

[95] Brenner S (1974). The genetics of *Caenorhabditis elegans*.. Genetics.

[96] Bean CJ, Schaner CE, Kelly WG (2004). Meiotic pairing and imprinted X chromatin assembly in *Caenorhabditis elegans*.. Nat Genet.

[97] Bilinski SM, Jaglarz MK, Szymanska B, Etkin LD, Kloc M (2004). Sm proteins, the constituents of the spliceosome, are components of nuage and mitochondrial cement in *Xenopus* oocytes.. Exp Cell Res.

[98] Dostie J, Mourelatos Z, Yang M, Sharma A, Dreyfuss G (2003). Numerous microRNPs in neuronal cells containing novel microRNAs.. RNA.

[99] Hillier LW, Reinke V, Green P, Hirst M, Marra MA (2009). Massively parallel sequencing of the polyadenylated transcriptome of *C. elegans*.. Genome Res.

[100] Townend J (2002). Practical statistics for environmental and biological scientists.

[101] Gunawardena S, Goldstein LS (2001). Disruption of axonal transport and neuronal viability by amyloid precursor protein mutations in *Drosophila*.. Neuron.

[102] Cao YJ, Dreixler JC, Couey JJ, Houamed KM (2002). Modulation of recombinant and native neuronal SK channels by the neuroprotective drug riluzole.. Eur J Pharmacol.

[103] Bensimon G, Lacomblez L, Meininger V (1994). A controlled trial of riluzole in amyotrophic lateral sclerosis. ALS/Riluzole Study Group.. N Engl J Med.

[104] Couratier P, Sindou P, Esclaire F, Louvel E, Hugon J (1994). Neuroprotective effects of riluzole in ALS CSF toxicity.. Neuroreport.

[105] Haddad H, Cifuentes-Diaz C, Miroglio A, Roblot N, Joshi V (2003). Riluzole attenuates spinal muscular atrophy disease progression in a mouse model.. Muscle Nerve.

[106] Nishimura AL, Mitne-Neto M, Silva HC, Richieri-Costa A, Middleton S (2004). A mutation in the vesicle-trafficking protein VAPB causes late-onset spinal muscular atrophy and amyotrophic lateral sclerosis.. Am J Hum Genet.

[107] Shen V, Liu H, Liu SW, Jiao X, Kiledjian M (2008). DcpS scavenger decapping enzyme can modulate pre-mRNA splicing.. RNA.

[108] Singh J, Salcius M, Liu SW, Staker BL, Mishra R (2008). DcpS as a therapeutic target for spinal muscular atrophy.. ACS Chem Biol.

[109] Nonis D, Schmidt MH, van de Loo S, Eich F, Dikic I (2008). Ataxin-2 associates with the endocytosis complex and affects EGF receptor trafficking.. Cell Signal.

[110] Lim J, Hao T, Shaw C, Patel AJ, Szabo G (2006). A protein-protein interaction network for human inherited ataxias and disorders of Purkinje cell degeneration.. Cell.

[111] Li S, Armstrong CM, Bertin N, Ge H, Milstein S (2004). A map of the interactome network of the metazoan *C. elegans*.. Science.

[112] Jeong H, Then F, Melia TJ, Mazzulli JR, Cui L (2009). Acetylation targets mutant huntingtin to autophagosomes for degradation.. Cell.

[113] Jia K, Hart AC, Levine B (2007). Autophagy genes protect against disease caused by polyglutamine expansion proteins in *Caenorhabditis elegans*.. Autophagy.

[114] Serra HG, Duvick L, Zu T, Carlson K, Stevens S (2006). RORalpha-mediated Purkinje cell development determines disease severity in adult SCA1 mice.. Cell.

[115] Petrelli A, Gilestro GF, Lanzardo S, Comoglio PM, Migone N (2002). The endophilin-CIN85-Cbl complex mediates ligand-dependent downregulation of c-Met.. Nature.

[116] Kaneko T, Maeda A, Takefuji M, Aoyama H, Nakayama M (2005). Rho mediates endocytosis of epidermal growth factor receptor through phosphorylation of endophilin A1 by Rho-kinase.. Genes Cells.

[117] Guillaumond F, Dardente H, Giguere V, Cermakian N (2005). Differential control of Bmal1 circadian transcription by REV-ERB and ROR nuclear receptors.. J Biol Rhythms.

[118] Forman BM, Chen J, Blumberg B, Kliewer SA, Henshaw R (1994). Cross-talk among ROR alpha 1 and the Rev-erb family of orphan nuclear receptors.. Mol Endocrinol.

[119] Freeman JL, Pitcher JA, Li X, Bennett V, Lefkowitz RJ (2000). alpha-Actinin is a potent regulator of G protein-coupled receptor kinase activity and substrate specificity in vitro.. FEBS Lett.

[120] Chatellard-Causse C, Blot B, Cristina N, Torch S, Missotten M (2002). Alix (ALG-2-interacting protein X), a protein involved in apoptosis, binds to endophilins and induces cytoplasmic vacuolization.. J Biol Chem.

[121] Lu L, Zhang Q, Timofeyev V, Zhang Z, Young JN (2007). Molecular coupling of a Ca2+-activated K+ channel to L-type Ca2+ channels via alpha-actinin2.. Circ Res.

[122] Chen B, Borinstein SC, Gillis J, Sykes VW, Bogler O (2000). The glioma-associated protein SETA interacts with AIP1/Alix and ALG-2 and modulates apoptosis in astrocytes.. J Biol Chem.

[123] Schmidt MH, Hoeller D, Yu J, Furnari FB, Cavenee WK (2004). Alix/AIP1 antagonizes epidermal growth factor receptor downregulation by the Cbl-SETA/CIN85 complex.. Mol Cell Biol.

[124] Carman CV, Lisanti MP, Benovic JL (1999). Regulation of G protein-coupled receptor kinases by caveolin.. J Biol Chem.

[125] Ralser M, Albrecht M, Nonhoff U, Lengauer T, Lehrach H (2005). An integrative approach to gain insights into the cellular function of human ataxin-2.. J Mol Biol.

[126] Lessing D, Bonini NM (2008). Polyglutamine genes interact to modulate the severity and progression of neurodegeneration in *Drosophila*.. PLoS Biol.

[127] Ciosk R, DePalma M, Priess JR (2004). ATX-2, the *C. elegans* ortholog of ataxin 2, functions in translational regulation in the germline.. Development.

[128] Satterfield TF, Pallanck LJ (2006). Ataxin-2 and its *Drosophila* homolog, ATX2, physically assemble with polyribosomes.. Hum Mol Genet.

[129] Nonhoff U, Ralser M, Welzel F, Piccini I, Balzereit D (2007). Ataxin-2 interacts with the DEAD/H-box RNA helicase DDX6 and interferes with P-bodies and stress granules.. Mol Biol Cell.

[130] Kozlov G, Trempe JF, Khaleghpour K, Kahvejian A, Ekiel I (2001). Structure and function of the C-terminal PABC domain of human poly(A)-binding protein.. Proc Natl Acad Sci U S A.

[131] Ikezu T, Trapp BD, Song KS, Schlegel A, Lisanti MP (1998). Caveolae, plasma membrane microdomains for alpha-secretase-mediated processing of the amyloid precursor protein.. J Biol Chem.

[132] Massenet S, Pellizzoni L, Paushkin S, Mattaj IW, Dreyfuss G (2002). The SMN complex is associated with snRNPs throughout their cytoplasmic assembly pathway.. Mol Cell Biol.

[133] Gareus R, Di Nardo A, Rybin V, Witke W (2006). Mouse profilin 2 regulates endocytosis and competes with SH3 ligand binding to dynamin 1.. J Biol Chem.

[134] Schafer DA, Weed SA, Binns D, Karginov AV, Parsons JT (2002). Dynamin2 and cortactin regulate actin assembly and filament organization.. Curr Biol.

[135] Witke W, Podtelejnikov AV, Di Nardo A, Sutherland JD, Gurniak CB (1998). In mouse brain profilin I and profilin II associate with regulators of the endocytic pathway and actin assembly.. EMBO J.

[136] Al-Ramahi I, Perez AM, Lim J, Zhang M, Sorensen R (2007). dAtaxin-2 mediates expanded Ataxin-1-induced neurodegeneration in a *Drosophila* model of SCA1.. PLoS Genet.

[137] Ghosh-Roy A, Desai BS, Ray K (2005). Dynein light chain 1 regulates dynamin-mediated F-actin assembly during sperm individualization in *Drosophila*.. Mol Biol Cell.

[138] Navarro-Lerida I, Martinez Moreno M, Roncal F, Gavilanes F, Albar JP (2004). Proteomic identification of brain proteins that interact with dynein light chain LC8.. Proteomics.

[139] Pan S, Wang R, Zhou X, He G, Koomen J (2006). Involvement of the conserved adaptor protein Alix in actin cytoskeleton assembly.. J Biol Chem.

[140] Gaillard S, Bartoli M, Castets F, Monneron A (2001). Striatin, a calmodulin-dependent scaffolding protein, directly binds caveolin-1.. FEBS Lett.

[141] Khandjian EW, Huot ME, Tremblay S, Davidovic L, Mazroui R (2004). Biochemical evidence for the association of fragile X mental retardation protein with brain polyribosomal ribonucleoparticles.. Proc Natl Acad Sci U S A.

[142] Gagne JP, Bonicalzi ME, Gagne P, Ouellet ME, Hendzel MJ (2005). Poly(ADP-ribose) glycohydrolase is a component of the FMRP-associated messenger ribonucleoparticles.. Biochem J.

[143] Castets F, Bartoli M, Barnier JV, Baillat G, Salin P (1996). A novel calmodulin-binding protein, belonging to the WD-repeat family, is localized in dendrites of a subset of CNS neurons.. J Cell Biol.

[144] Shao J, Welch WJ, Diprospero NA, Diamond MI (2008). Phosphorylation of profilin by ROCK1 regulates polyglutamine aggregation.. Mol Cell Biol.

[145] Baillat G, Gaillard S, Castets F, Monneron A (2002). Interactions of phocein with nucleoside-diphosphate kinase, Eps15, and Dynamin I.. J Biol Chem.

[146] Sittler A, Walter S, Wedemeyer N, Hasenbank R, Scherzinger E (1998). SH3GL3 associates with the Huntingtin exon 1 protein and promotes the formation of polygln-containing protein aggregates.. Mol Cell.

[147] Qin ZH, Wang Y, Sapp E, Cuiffo B, Wanker E (2004). Huntingtin bodies sequester vesicle-associated proteins by a polyproline-dependent interaction.. J Neurosci.

[148] Fallon L, Belanger CM, Corera AT, Kontogiannea M, Regan-Klapisz E (2006). A regulated interaction with the UIM protein Eps15 implicates parkin in EGF receptor trafficking and PI(3)K-Akt signalling.. Nat Cell Biol.

[149] Salcini AE, Hilliard MA, Croce A, Arbucci S, Luzzi P (2001). The Eps15 *C. elegans* homologue EHS-1 is implicated in synaptic vesicle recycling.. Nat Cell Biol.

[150] Krishnan KS, Rikhy R, Rao S, Shivalkar M, Mosko M (2001). Nucleoside diphosphate kinase, a source of GTP, is required for dynamin-dependent synaptic vesicle recycling.. Neuron.

[151] Sharma A, Lambrechts A, Hao le T, Le TT, Sewry CA (2005). A role for complexes of survival of motor neurons (SMN) protein with gemins and profilin in neurite-like cytoplasmic extensions of cultured nerve cells.. Exp Cell Res.

[152] Giesemann T, Rathke-Hartlieb S, Rothkegel M, Bartsch JW, Buchmeier S (1999). A role for polyproline motifs in the spinal muscular atrophy protein SMN. Profilins bind to and colocalize with smn in nuclear gems.. J Biol Chem.

[153] Bowerman M, Shafey D, Kothary R (2007). Smn depletion alters profilin II expression and leads to upregulation of the RhoA/ROCK pathway and defects in neuronal integrity.. J Mol Neurosci.

[154] Reeve SP, Bassetto L, Genova GK, Kleyner Y, Leyssen M (2005). The *Drosophila* fragile X mental retardation protein controls actin dynamics by directly regulating profilin in the brain.. Curr Biol.

[155] Tessier CR, Broadie K (2008). *Drosophila* fragile X mental retardation protein developmentally regulates activity-dependent axon pruning.. Development.

