# Supplementary material for: Conserved Genes Act as Modifiers of Invertebrate SMN Loss of Function Defects
Source: PLoS Genet. 2010 Oct 28;6(10):e1001172. doi: 10.1371/journal.pgen.1001172 (PMC2965752; doi:10.1371/journal.pgen.1001172)
Supplement: Table S1 — Summary of C. elegans growth assays. (0.09 MB DOC) [file pgen.1001172.s001.doc]

| **Table S1. Summary of *C. elegans* growth assays** | | | | |  |  |  |
| --- | --- | --- | --- | --- | --- | --- | --- |
|  |  |  |  |  |  |  |  |
| *Dm* gene | *Ce* gene | *Cesmn-1(lf)* homozygous | | | *+/Cesmn-1(lf)* heterozygous | | |
|  | RNAi target | Mean | SEM | p | Mean | SEM | p |
| - | *none* | 18.0 | 2.2 | - | 46.1 | 1.0 | - |
| SMN | *smn-1* | 5.9 | 4.4 | <0.001 | 31.2 | 1.1 | 0.017 |
| Fim | *plst-1* | 15.2 | 3.2 | 0.390 | 55.2 | 2.5 | 0.023 |
| actinin | *atn-1* | 22.5 | 4.1 | 0.029 | 42.8 | 1.0 | 0.763 |
| Usp | *nhr-25* | 16.8 | 4.9 | 0.815 | 37.5 | 3.6 | 0.159 |
| SK | *kcnl-2* | 12.2 | 1.1 | 0.011 | 41.5 | 1.1 | 0.280 |
| CG32796 | *sax-3* | 14.9 | 5.1 | 0.185 | 42.2 | 2.8 | 0.763 |
| Trol | *unc-52* | 16.8 | 3.5 | 0.815 | 46.6 | 2.8 | 0.651 |
| Sprint | *C48G7.2* | 15.9 | 5.5 | 0.483 | 48.0 | 4.1 | 0.547 |
| CG6414 | *-* | - | - | - | - | - | - |
| CG33172 | *cash-1* | 26.1 | 5.4 | 0.876 | 64.1 | 10.9 | 0.021 |
| CG1835 | *-* | - | - | - | - | - | - |
| CG18375 | *ape-1* | 10.7 | 8.6 | 0.024 | 49.5 | 2.0 | 0.841 |
| CG34379 | *-* | - | - | - | - | - | - |
| CG8589 | *-* | - | - | - | - | - | - |
| CG11450 | *hlh-4* | - | - | - | - | - | - |
| Wit | *sma-6* | 15.1 | 6.2 | 0.213 | 51.5 | 2.3 | 0.393 |
| Wit | *daf-4* | 19.0 | 8.0 | 0.938 | 46.9 | 5.3 | 0.920 |
| CG5361 | *-* | - | - | - | - | - | - |
| Fmr1 | *ZK418.9* | 18.1 | 3.5 | 0.756 | 41.8 | 2.3 | 0.340 |
| Eip75B | *nhr-85* | 7.9 | 5.2 | 0.002 | 44.2 | 1.3 | 0.832 |
| CG1927 | *-* | - | - | - | - | - | - |
| Btl | *egl-15* | 8.9 | 5.4 | 0.004 | 44.5 | 1.4 | 0.655 |
| Raptor | *daf-15* | 15.9 | 3.2 | 0.679 | 45.6 | 3.6 | 0.920 |
| Mipp2 | *pho-5* | 17.2 | 1.9 | 0.938 | 53.6 | 3.0 | 0.079 |
| Nep1 | *nep-2* | 15.5 | 5.9 | 0.586 | 35.6 | 3.1 | 0.159 |
| Moesin | *erm-1* | 14.1 | 1.9 | 0.392 | 45.1 | 4.0 | 0.725 |
| Nek2 | *nekl-3* | 13.2 | 2.8 | 0.031 | 43.2 | 1.5 | 0.709 |
| p115 | *uso-1* | 6.9 | 4.4 | 0.002 | 43.5 | 0.5 | 0.766 |
| ctp | *dlc-1* | 14.8 | 7.7 | 0.131 | 66.4 | 2.8 | 0.039 |
| Rho-4 | *rom-1* | 14.5 | 3.8 | 0.161 | 51.0 | 2.6 | 0.191 |
| CG1561 | *-* | - | - | - | - | - | - |
| CG3136 | *atf-6* | 10.5 | 7.4 | 0.010 | 40.0 | 0.4 | 0.421 |
| CG8920 | *C56G2.1* | 15.8 | 1.7 | 0.312 | 43.6 | 1.1 | 0.801 |
| CG13868 | *-* | - | - | - | - | - | - |
| CG12214 | *C52B9.3* | 16.6 | 2.6 | 0.697 | 44.5 | 4.1 | 0.920 |
| CG13775 | *C53D6.3* | 14.3 | 5.6 | 0.120 | 42.5 | 0.2 | 0.763 |
| CG10561 | *C24C6.6* | 21.2 | 1.8 | 0.312 | 49.5 | 1.3 | 0.248 |
|  |  |  |  |  |  |  |  |
| *Dm* gene | *Ce* gene | *Cesmn-1(lf)* homozygous | | | *+/Cesmn-1(lf)* heterozygous | | |
|  | RNAi target | Mean | SEM | p | Mean | SEM | p |
| - | *none* | 24.0 | 3.5 | - | 42.2 | 2.9 | - |
| CG4325 | *B0432.13* | 25.3 | 1.8 | 0.629 | 37.8 | 3.1 | 0.393 |
| CG17323 | *ugt-49* | 10.7 | 3.6 | <0.001 | 19.5 | 9.4 | 0.007 |
| CG17322 | *-* | - | - | - | - | - | - |
| CG17324 | *-* | - | - | - | - | - | - |
| CG11200 | *dhs-22* | 22.5 | 2.1 | 1.000 | 39.5 | 7.2 | 1.000 |

Table S1 is divided by horizontal lines into three sections corresponding to potential *Cesmn-1(lf)* modifier genes originally identified in humans and *Drosophila* (1 in *Hs* and 40 in *Dm*). The *Drosophila* gene and *C. elegans* ortholog targeted by RNAi are listed in the first two columns of the table. The 3^rd^ and 4^th^ columns give the average size and standard error of the mean (S.E.M.) for *Cesmn-1(lf)* homozygous animals. The significance *versus* empty vector RNAi was determined for each gene using the two-tailed Mann-Whitney *U* test or Chi-squared test according to sample-specific parameters and is reported in column 5. Columns 6 through 8 report the results of the same analysis for *+/Cesmn-1(lf)* heterozygous animals. The *hlh-4(RNAi)* clone in the feeding library was incorrect; see Materials and Methods for details. Significant p-values (p<0.05) are shaded. The bottom panel of Table S1 reports data from a separate experiment conducted after the COPAS Biosorter was re-calibrated, accounting for the small discrepancies between controls.
