# Supplementary material for: Conserved Genes Act as Modifiers of Invertebrate SMN Loss of Function Defects
Source: PLoS Genet. 2010 Oct 28;6(10):e1001172. doi: 10.1371/journal.pgen.1001172 (PMC2965752; doi:10.1371/journal.pgen.1001172)
Supplement: Table S2 — Summary of C. elegans pharyngeal pumping assays. (0.09 MB DOC) [file pgen.1001172.s002.doc]

| **Table S2. Summary of *C. elegans* pharyngeal pumping assays** | | | | |  |  |  |
| --- | --- | --- | --- | --- | --- | --- | --- |
|  |  |  |  |  |  |  |  |
| *Drosophila* gene | *C.elegans* gene RNAi target | Mean+SEM *gene(RNAi)* | Mean+SEM *empty(RNAi)* | P-value | % change | Mean % (by trials) | Chi Square P (by trials) |
| SMN | *C41G7.1 (smn-1)* | 26.8±6.2 | 53.0±9.2 | 0.023 | 50 | 56 | <0.001 |
| Fim | *Y104H12BR.1 (plst-1)* | 43.3±8.4 | 53.0±9.2 | 0.415 | 82 | 86 | <0.001 |
| actinin | *W04D2.1 (atn-1)* | 41.3±8.4 | 41.3±5.8 | >0.999 | 100 | 99 | - |
| Usp | *F11C1.6 (nhr-25)* | 66.7±11.0 | 48.3±8.1 | 0.653 | 138 | 155 | <0.001 |
| SK | *F08A10.1 (kcnl-2)* | 48.1±8.0 | 42.0±7.0 | 0.685 | 115 | 147 | <0.001 |
| CG32796 | *ZK377.2 (sax-3)* | 43.3±7.1 | 56.5±11.4 | 0.328 | 77 | 79 | - |
| Trol | *ZC101.2 (unc-52)* | 31.1±7.2 | 39.3±5.7 | 0.054 | 79 | 78 | 0.287 |
| Sprint | *C48G7.2* | 34.5±5.9 | 32.5±9.7 | 0.176 | 106 | 111 | - |
| CG6414 | *-* | - | - | - | - | - | - |
| CG33172 | *K07C5.8 (cash-1)* | 32.3±4.9 | 30.6±5.1 | 0.791 | 105 | 116 | <0.001 |
| CG1835 | *-* | - | - | - | - | - | - |
| CG18375 | *F46F3.4 (ape-1)* | 47.8±6.9 | 46.9±8.1 | 0.889 | 102 | 102 | <0.001 |
| CG34379 | *T09A5.10 (lin-5)* | 37.8±9.7 | 33.8±7.3 | 0.512 | 112 | 111 | - |
| CG8589 | *-* | - | - | - | - | - | - |
| CG11450 | *T05G5.2 (hlh-4)* | - | - | - | - | - | - |
| Wit | *F29C4.1 (sma-6)* | 42.0±9.8 | 32.5±9.7 | 0.332 | 129 | 128 | - |
| Wit | *C05D2.1 (daf-4)* | 26.4±6.5 | 38.2±6.8 | 0.031 | 69 | 70 | <0.001 |
| CG5361 | *-* | - | - | - | - | - | - |
| Fmr1 | *ZK418.9* | 48.5±11.2 | 33.8±7.3 | 0.275 | 144 | 146 | - |
| Eip75B | *W05B5.3 (nhr-85)* | 62.6±9.9 | 55.8±9.3 | 0.425 | 112 | 115 | 0.806 |
| CG1927 | *-* | - | - | - | - | - | - |
| Btl | *F58A3.2 (egl-15)* | 46.6±13.9 | 62.2±11.5 | 0.168 | 75 | 80 | - |
| Raptor | *C10C5.6 (daf-15)* | 52.1±10.2 | 62.2±11.5 | 0.515 | 84 | 89 | - |
| Mipp2 | *B0361.7 (pho-5)* | 34.8±8.3 | 33.8±7.3 | 0.958 | 103 | 102 | - |
| Nep1 | *T05A8.4 (nep-2)* | 30.0±7.6 | 41.3±5.8 | 0.876 | 73 | 74 | - |
| Moesin | *C01G8.6 (erm-1)* | 26.3±6.3 | 32.7±7.0 | 0.219 | 80 | 91 | - |
| Nek2 | *F19H6.1 (nekl-3)* | 62.5±11.0 | 62.2±11.5 | 0.986 | 100 | 108 | - |
| p115 | *K09B11.9 (uso-1)* | 30.8±8.8 | 32.5±9.7 | 0.507 | 95 | 92 | - |
| Ctp | *T26A5.9 (dlc-1)* | 56.3±13.9 | 71.5±15.2 | 0.491 | 79 | 78 | - |
| Rho-4 | *F26F4.3 (rom-1)* | 48.3±8.7 | 62.2±11.5 | 0.340 | 78 | 81 | - |
| CG1561 | *-* | - | - | - | - | - | - |
| CG3136 | *F45E6.2 (atf-6)* | 31.9±7.3 | 32.7±7.0 | 0.465 | 97 | 107 | - |
| CG8920 | *C56G2.1* | 65.8±9.9 | 56.5±11.4 | 0.543 | 116 | 127 | - |
| CG13868 | *-* | - | - | - | - | - | - |
| CG12214 | *C52B9.3* | 38.3±9.5 | 50.6±12.1 | 0.906 | 76 | 78 | - |
| CG13775 | *C53D6.6* | 40.8±9.7 | 41.3±5.8 | 0.965 | 99 | 98 | - |
| CG10561 | *C24G6.6* | 40.5±8.2 | 50.6±12.1 | 0.914 | 80 | 79 | - |
|  |  |  |  |  |  |  |  |
| *Drosophila* gene | *C.elegans* gene RNAi target | Mean+SEM *gene(RNAi)* | Mean+SEM *empty(RNAi)* | P-value | % change | Mean % (by trials) | Chi Square P (by trials) |
| CG4325 | *B0432.13* | 30.0+13.1 | 23.6+11.0 | 0.925 | 126.7 | 130.8 | 0.711 |
| CG17323 | *AC3.2 (ugt-49)* | 41.4+14.8 | 33+10.8 | 0.928 | 79.7 | 148.5 | 0.017 |
| CG17322 | - | - | - | - | - | - | - |
| CG17324 | - | - | - | - | - | - | - |
| CG11200 | *C15H11.4 (dhs-22)* | 24.9+9.7 | 23.68+11.0 | 0.623 | 105.2 | 133.5 | 0.087 |

Table S2 is divided into three sections corresponding to SMN modifier genes originally identified in humans and *Drosophila* (1 in *Hs* and 40 in *Dm*). The *Drosophila* gene and *C. elegans* ortholog targeted by RNAi are listed in the first two columns of the table. The 3^rd^ and 4^th^ columns give the mean pumping rate and standard error of the mean (S.E.M.) for *Cesmn-1(lf)* animals in all trials. The significance *versus* empty vector RNAi was determined for each gene using an unpaired two-sample *t*-test or a Mann-Whitney *U* two-tailed test according to sample-specific parameters and is reported in column 5. The percentage change in *Cesmn-1(lf)* pumping rates reported in column 6 equals (100 x pumping rate of *Cesmn-1(lf)*/rate of *+/Cesmn-1(lf)*). As pumping rates of control animals varied from day to day (trial to trial) because of food density and other variables, the percent change in *Cesmn-1(lf)* pumping rates was also calculated for each independent trial (n>10 animals each trial, column 7). Each gene was examined in at least two trials. If either of these two trials yielded a significant change in pumping rates, then a total of four independent trials was undertaken and a p-value was determined by Chi-square analysis (column 8). Significant p-values (p<0.05) are shaded. The *hlh-4(RNAi)* clone in the feeding library was incorrect; see Materials and Methods for details. Only the results of RNAi knockdown studies are reported here; double mutant analysis is reported in Figures 3 and 4. The bottom panel of Table S2 refers to a separate experiment conducted.
