# Supplementary material for: Conserved Genes Act as Modifiers of Invertebrate SMN Loss of Function Defects
Source: PLoS Genet. 2010 Oct 28;6(10):e1001172. doi: 10.1371/journal.pgen.1001172 (PMC2965752; doi:10.1371/journal.pgen.1001172)
Supplement: Table S3 — Invertebrate modifier genes specifically affect Cesmn-1(lf) pharyngeal pumping defects. (0.05 MB DOC) [file pgen.1001172.s003.doc]

| **Table S3. Invertebrate modifier genes specifically affect *Cesmn-1(lf)* pharyngeal pumping defects** | | | | | | | |  |
| --- | --- | --- | --- | --- | --- | --- | --- | --- |
|  |  |  |  |  |  |  |  | |
| *Drosophila* gene | *C.elegans* gene  RNAi target | Mean+SEM *gene(RNAi)* | Mean+SEM *empty(RNAi)* | P-value |  |  |  | |
| SMN | *C41G7.1 (smn-1)* | 113.7+12.3 | 132+13.3 | 0.413 |  |  |  | |
| Fim | *Y104H12BR.1 (plst-1)* | 124.2+12.8 | 102.9+12.4 | 0.231 |  |  |  | |
| Actinin | *W04D2.1 (atn-1)* | 126.3+14.8 | 123.5+16.8 | 0.791 |  |  |  | |
| Usp | *F11C1.6 (nhr-25)* | 116.4+11.8 | 102.9+12.4 | 0.445 |  |  |  | |
| SK | *F08A10.1 (kcnl-2)* | 95.4+12.9 | 102.9+12.4 | 0.429 |  |  |  | |
| CG33172 | *K07C5.8 (cash-1)* | 110.7+14.7 | 97.8+9.3 | 0.660 |  |  |  | |
| CG18375 | *F46F3.4 (ape-1)* | 148.2+16.0 | 109.5+15.1 | 0.071 |  |  |  | |
| Wit | *C05D2.1 (daf-4)* | 130.2+14.2 | 102.9+12.4 | 0.201 |  |  |  | |
| Eip75B | *W05B5.3 (nhr-85)* | 92.1+18.1 | 109.5+15.1 | 0.370 |  |  |  | |
| Btl | *F58A3.2 (egl-15)* | 90.9+15.2 | 109.5+15.1 | 0.368 |  |  |  | |
| Nek2 | *F19H6.1 (nekl-3)* | 155.1+18.6 | 109.5+15.1 | 0.056 |  |  |  | |
| p115 | *K09B11.9 (uso-1)* | 92.8+17.2 | 102.9+12.4 | 0.395 |  |  |  | |
| Ctp | *T26A5.9 (dlc-1)* | 118.5+14.3 | 123.5+16.8 | 0.857 |  |  |  | |
| CG3136 | *F45E6.2 (atf-6)* | 110.1+18.2 | 109.5+15.1 | 0.989 |  |  |  | |
| CBC20 | *F26A3.2 (ncbp-2)* | 113.1+16.9 | 123.5+16.8 | 0.707 |  |  |  | |
| Gprk | *W02B3.2 (grk-2)* | 135.3+12.4 | 132+13.3 | 0.659 |  |  |  | |
| FMRF | *C18D1.3 (flp-4)* | 118.5+12.5 | 132+13.3 | 0.398 |  |  |  | |
| none | *T02G5.3* | 119.1+11.9 | 132+13.3 | 0.603 |  |  |  | |

To address the specificity of cross-species *Cesmn-1(lf)* modifier genes, the impact of their RNAi knockdown on an unrelated pharyngeal pumping defective strain was examined. Loss of *egl-30* (G_q_α) gene function decreases pharyngeal pumping rates [74]. RNAi knockdown of the *Cesmn-1(lf)* modifiers listed in Table 4 did not significantly alter the pharyngeal pumping rates of *egl-30(ad805)* animals, suggesting that these genes do not act non-specifically. *egl-30(ad805)* animals are sensitive to RNAi knockdown by feeding based on *bli-1(RNAi)* and *dpy-11(RNAi)* results (data not shown). Pharyngeal pumping was assessed in at least two independent trials (n>10 animals each trial). Statistical analysis was performed using the Mann-Whitney *U* two-tailed test.
