# Supplementary material for: Conserved Genes Act as Modifiers of Invertebrate SMN Loss of Function Defects
Source: PLoS Genet. 2010 Oct 28;6(10):e1001172. doi: 10.1371/journal.pgen.1001172 (PMC2965752; doi:10.1371/journal.pgen.1001172)
Supplement: Table S4 — Body length determinations for Cesmn-1(lf) modifier genes. (0.05 MB DOC) [file pgen.1001172.s004.doc]

| **Table S4. Body length determinations for *Cesmn-1(lf)* modifier genes** | | | | |  | |  |
| --- | --- | --- | --- | --- | --- | --- | --- |
|  |  |  |  |  | |  |  |
| *Ce* RNAi target | *Cesmn-1(lf)* Mean Body Length (cm) | P-value |  |  | |  |  |
| none | 17.9+0.2 | - |  |  | |  |  |
| *ncbp-2* | 16.0+0.5 | <0.001 |  |  | |  |  |
| *flp-4* | 19.1+0.2 | <0.001 |  |  | |  |  |
| *grk-2* | 17.1+0.5 | 0.508 |  |  | |  |  |
| *T02G5.3* | 17.1+0.6 | 0.302 |  |  | |  |  |
|  |  |  |  |  | |  |  |
| *Ce* RNAi target | Mean Body Length (cm) | P-value |  |  | |  |  |
| none | 28.3+0.4 | - |  |  | |  |  |
| *plst-1* | 29.6+0.3 | 0.018 |  |  | |  |  |
|  |  |  |  |  | |  |  |
| *Ce* genotype | Mean Body Length (cm) | P-value *vs. +/Cesmn-1(lf)* | P-value |  | |  |  |
| *+/Cesmn-1(lf)* | 27.0+0.4 | - |  |  | |  |  |
| *+/Cesmn-1(lf);plst-1(lf)* | 25.5+0.5 | 0.032 |  |  | |  |  |
| *plst-1(lf)* | 29.1+0.3 | <0.001 | <0.001 | *vs. +/Cesmn-1(lf);plst-1* | | | |
| *Cesmn-1(lf)* | 16.9+0.4 | <0.001 |  |  | |  |  |
| *Cesmn-1(lf);plst-1(lf)* | 17.4+0.2 | <0.001 | 0.363 | *vs. Cesmn1(lf)* | | |  |

*Cesmn-1(lf)* homozygous animals (top panel), wild type (N2) animals (middle panel), *Cesmn-1(lf);plst-1(lf)* double mutant and corresponding control animals were reared on bacterial RNAi feeding strains or empty control vector as indicated (column 1). The mean body length of each genotype was determined at day 3 post-hatching in at least two independent trials (total ≥15). *ncbp-2* and *flp-4* RNAi knockdown significantly reduced and increased the average body length of *Cesmn-1(lf)* animals, respectively, in relation to age-matched controls (top panel). Wild type animals were significantly longer in size when reared on *plst-1(RNAi)* (middle panel). In the bottom panel, *plst-1* loss of function young adult animals are longer than *+/Cesmn-1(lf)* animals or *+/Cesmn-1(lf);plst-1(lf)* animals. However, double mutant *Cesmn-1(lf);plst-1(lf)* young adult animals are not significantly different in body length than *Cesmn-1(lf)* homozygous animals. See Materials and Methods (Text S1) for details. The two-tailed Mann-Whitney *U* test (p<0.05) was used for statistical analysis; significant changes are shaded.
