# Supplementary material for: Conserved Genes Act as Modifiers of Invertebrate SMN Loss of Function Defects
Source: PLoS Genet. 2010 Oct 28;6(10):e1001172. doi: 10.1371/journal.pgen.1001172 (PMC2965752; doi:10.1371/journal.pgen.1001172)
Supplement: Text S1 — Methods for supplementary files. (0.02 MB DOC) [file pgen.1001172.s005.doc]

**Materials and Methods (supplementary data)**

Body Length Assay

Animals reared on bacterial RNAi feeding strains and/or control empty vector were placed on cold (+4°C) standard *C. elegans* culture plates to straighten animals for imaging. Images were captured on a Zeiss Discovery V20 at 130X magnification by using Axiovision Rel. 4.6 application. At least 10 animals of each genotype were scored for their body length size in at least two independent trials (total n≥15). Images were printed, and body length was measured, blinded as to genotype/treatment utilizing an Alvin Truflex Flexible Curve ruler.
