# Supplementary material for: Conserved Genes Act as Modifiers of Invertebrate SMN Loss of Function Defects
Source: PLoS Genet. 2010 Oct 28;6(10):e1001172. doi: 10.1371/journal.pgen.1001172 (PMC2965752; doi:10.1371/journal.pgen.1001172)
Supplement: Text S2 — Supplementary discussion of invertebrate modifier genes. (0.33 MB DOC) [file pgen.1001172.s006.doc]

**Supplemental discussion of modifier gene function**

***ncbp-2*** encodes the *C. elegans* Cap Binding Protein 20 (CBP20 or Cbp20) ortholog [1]. Vertebrate CBP20 and CBP80 assemble to form the cap-binding complex that binds to the 5’ end of mRNAs during nuclear export [2]. The CBC complex is replaced by the eIF4E complex after the first round of translation and interacts with numerous proteins. In addition to this canonical role, CBC also inhibits transcript deadenylation, plays a role in U snRNA export, nonsense mediated decay, and is required for miRNA maturation [3-8]. Human CBC antibodies recognize the *C. elegans* NCBP-2 protein that is found in the nucleus and in cytoplasmic puncta of gonads and oocytes [1]. Mammalian DcpS displaces CBC20 from mRNA cap structures and loss of DcpS may result in accumulation of CBC20 bound to 5’ cap structures with consequent aberrant first intron splicing [9]. A recent study found that inhibition of mRNA 5’ decapping enzyme DcpS by C5-substituted quinazolines increases SMN2 transcription potentially linking DcpS to SMN [10].

***T02G5.3*** encodes a gene of unknown function. This gene has not been previously characterized and no clear orthologs exist outside the *Caenorhabditis* genus.

***grk-2*** is one of two *C. elegans* genes encoding G-protein coupled receptor kinases (GRK) [11]. *C. elegans* *grk-2* is expressed in neurons and has been previously implicated in sensory response and alpha-synuclein toxicity [11,12]. Classical studies in vertebrates have demonstrated a role for GRK proteins in inactivation and internalization of beta-adrenergic receptors following ligand-activation, but recent studies have implicated GRKs in numerous other processes. Grk2 and FMRP, the Fragile X Mental Retardation Protein, co-localize, co-precipitate and regulate dopaminergic receptors in mammalian neurons [13]. SMN has also been found in FMRP granules [14] suggesting a possible connection between local translation and SMN function. Vertebrate alpha-actinin directly binds and inhibits the function of mammalian GRK proteins suggesting a possible link between two *C. elegans* modifiers, *grk-2* and *atn-1* [15].

***flp-4*** is one of 33 *C. elegans* genes encoding FMRFamide family neuropeptides [16]. *C. elegans* *flp-4* is expressed in a small subset of neurons including I5, I6 and NSM pharyngeal neurons, but a role for *flp-4* have not been previously established in any behavior [17]. FMRFamide neuropeptides regulate diverse behaviors in invertebrates acting *via* synaptic and hormonal activation of neuropeptide gated chloride channels and G-protein coupled receptors [18,19]. Among the downstream targets of FMRFamide signaling are SK channels and post-synaptic L-type channels [20,21]

***uso-1*** encodes a protein orthologous to the vesicle docking protein p115, also known as Uso1 or transcytosis-associated protein (TAP), which acts in vesicle tethering during Rab1-associated, trans-Golgi transport [22-24]. In addition to classical apical/basal sorting pathways, p115 plays a role in transcytosis in which transmembrane proteins are secreted directly to the surface of the soma, undergo endocytosis, and then are targeted to the appropriate functional compartment. In neurons, transcytosis targets transmembrane proteins to distinct dendritic and/or axonal compartments [25-27] and helps to maintain the pre-synaptic localization of neurotransmitter receptors, NgCAM and other proteins [28-30].

***nhr-85*** encodes the *C. elegans* ortholog of Reverse Erb alpha (RevErb-alpha, a vertebrate nuclear hormone receptor that acts antagonistically to the closely related retinoic receptor alpha (RAR-alpha) in various transcriptional contexts including regulation of the Bmal1 ANRT-family transcription factor and, consequently, circadian rhythms [31-33]. The *Drosophila* ortholog of *nhr-85*, Eip75B, is developmentally regulated by ecdysone, a hormone that controls entry into pupal stages; Eip75B function may be regulated by nitric or carbon monoxide binding to a coordinated heme group [34-36].

***egl-15*** encodes a *C. elegans* FGF (Fibroblast Growth Factor) receptor. FGF signaling has been implicated in numerous intercellular signaling events including growth regulation, NMJ function and development [37-39]. FGF can regulate expression of circadian rhythm genes in vertebrate cells *via* Bmal phosphorylation [40]. It has also been suggested that *C. elegans* FGF ligand trafficking may depend on interaction with transcytosed LDL receptors [41,42]. SMN may be a target of FGF signaling as a nuclear isoform of the fibroblast growth factor FGF-2 competes with Gemin2 for binding to SMN [43].

***atf-6*** encodes the *C. elegans* ortholog of the activating transcription factor Atf6 [44]. Unfolded proteins, ER stress or oxidative insult results in cleavage of Atf6 associated with the endoplasmic reticulum (ER), nuclear translocation, and target gene transcription [45,46]. Atf6 is one of three major unfolded protein stress response pathways in vertebrates, acting in parallel with PERK/eIF2a and IRE pathways in cytoplasmic and, likely, dendritic endoplasmic reticulum [47]. Mammalian VAPB binds to and modulates the function of ATF6; VAPB is the disease gene associated with Amyotrophic Lateral Sclerosis VIII (ALS8) [48,49]. Afflicted individuals display either ALS symptoms or SMA-like symptoms in ALS8 families.

***ape-1*** is the likely *C. elegans* ortholog of *Drosophila* CG18375 and mammalian p53 binding protein, p53P2 , also known as iASPP. iASPP binds to and inhibits p53 activation under normal conditions preventing inappropriate apoptosis while affecting cell cycle arrest [50-54]. p53 is a major player in DNA damage response; p53 activation can activate autophagy and result in cell death in many contexts pertinent to neurodegeneration [55-58]. Diminishing *ape-1*/iASPP function may increase the odds of p53 activation and increase sensitivity of neurons or muscles to pro-apoptotic signals.

***nekl-3*** encodes the closest *C. elegans* ortholog of human NEK7 and was selected for analysis as a possible *C. elegans* ortholog of *Drosophila* Nek2 [59]. In vertebrates, NEK7 has been implicated in mitotic regulation acting with related NIMA kinases in centrosomal microtubule function and cytokinesis [60,61].

***atn-1*** encodes the *C. elegans* ortholog of alpha actinin [62]. Actinins, like plastins, are members of the actin-bundling, spectrin superfamily; they bind and cross-link cytoskeletal actin filaments (F-actin) with numerous consequences [63-65]. Interactions with F-actin are regulated by calcium binding to alpha-actinin EF-hand domains or by phosphotidylinositols in muscles. Mammalian alpha-actinin is found in muscle Z-discs/dense bodies, in neuronal post-synaptic densities and various adhesion sites in multiple tissues [66-70]. The cellular and subcellular distribution of *C. elegans* *atn-1* has not been completely defined, but *atn-1* is expressed in *C. elegans* pharyngeal and body muscles [71,72]. In vertebrates, alpha-actinin is found in complexes with numerous proteins including, Alix, the Raver1 RNA-binding protein, L-type calcium channels, calcium-activated potassium channels, and neurotransmitter receptors [73-79]. In *Drosophila* muscle tissue SMN has been found in a complex with alpha-actinin [80].

***cash-1*** encodes the *C. elegans* ortholog of mammalian striatin known as CKA in *Drosophila* [81]. Striatin contains calveolin-binding, calmodulin-binding, and WD-repeat domains that may couple endocytosis to signaling pathways and hormone receptors [82]. Striatin expression is highly enriched in the nervous system [83]. A recent study suggests that WD-repeats may function as RNA binding domains; it is possible that striatin also binds RNA [84].

***dlc-1*** encodes a dynein light chain similar to *Drosophila* cut up (ctp) [85]. Dynein plays numerous roles in intracellular trafficking including regulation of dynamin F-actin assembly during mRNA transport [86,87] and action with dynactin for transport of cargos on microtubule networks [88]. The dynein/dynactin complex plays a critical role in motorneuron retrograde transport of Gbb, a *Drosophila* TGF-beta ortholog [89], being consistent with the notion that the TGF-beta/Dpp pathway modifies SMN loss of function defects in *Drosophila* [90]. Other studies have found links between transport and neurodegenerative disease [91]. These include a recent study demonstrating that loss of *Drosophila* dynein light chain function perturbs autophagy and enhances neuromuscular synaptic and functional defects in a model of Spinal Bulbar Muscular Atrophy (SBMA) polyglutamine toxicity [92].

***kcnl-2*** likely encodes a *C. elegans* SK channel subunit. SK2 channels are small conductance potassium channels activated by calcium entry through voltage-gated calcium channels [93-95]. SK channels have relatively slow calcium influx currents that play a role in repolarization after depolarization and their loss generally leads to prolonged activation of neurons or muscles [95-98]. SK2 channels are post-synaptic in neurons and are important in after-hyperpolarization following action potentials [97-100] Loss of SK channel function in transgenic mice causes ataxia and neurodegeneration [101]. Riluzole is the only approved therapeutic treatment for ALS (amyotrophic lateral sclerosis) [102-104] Previous studies have suggested that actions of this drug include activation of SK channels [105]; riluzole ameliorates motor neuron synaptic defects in mice lacking SMN exon 7 and increases median survival [106]. While studies in mice were encouraging, the only published study addressing riluzole in SMA patients had insufficient power to address efficacy [106-108]. The validation of SK channels and ATF6 orthologs as cross-species modifiers of SMN loss of function defects and the ALS8 phenotypic spectrum suggests common molecular mechanisms in ALS and SMA [109]. SK2 channels interact directly with and are functionally modulated by aplha-actinin in muscle sarcomere Z-lines in cardiac myocytes. alpha-actinin also binds to L-type calcium channels functionally coupling these two channels [76,110]. SMN protein interacts with alpha-actinin and has been localized to the Z-lines of striated muscles of the body in *Drosophila* [80].

***nhr-25*** was selected for analysis as one of the two *C. elegans* proteins most similar to *Drosophila* Usp (Ultraspiracle) as no clear ortholog of Usp is found in the *C. elegans* genome. NHR-25 protein is most similar to mammalian SF-1 (steroidogenic factor-1) and *Drosophila* Ftz (fushi tarazu transcription factor 1) [33]. The extent of cross-species orthology in *C. elegans* will require functional analysis. Both *Drosophila* Ftz and Usp proteins play a role in ecdysone regulated molting and a role for *nhr-25* in molting has been proposed based on RNAi studies [33,111,112]. RXR-alpha is a retinoic acid activated nuclear hormone receptor that has many targets including directly activation of the TGF-beta downstream activator Smad2 in muscles [113,114]. *nhr-25* and *nhr-85* (described above) encode proteins in the nuclear hormone receptor superfamily whose likely vertebrate orthologs dimerize with RAR-alpha receptors [33]. A recent study finds that RXR-alpha predominantly localizes to the cytoplasm and processes of differentiating hippocampal neurons, unlike other RXR family members [113] suggesting that nuclear hormone receptor function in neuronal processes may be pertinent to SMA loss of function defects.

**References**

1. Lall S, Piano F, Davis RE (2005) Caenorhabditis elegans decapping proteins: localization and functional analysis of Dcp1, Dcp2, and DcpS during embryogenesis. Mol Biol Cell 16: 5880-5890.

2. Lewis JD, Izaurralde E (1997) The role of the cap structure in RNA processing and nuclear export. Eur J Biochem 247: 461-469.

3. Balatsos NA, Nilsson P, Mazza C, Cusack S, Virtanen A (2006) Inhibition of mRNA deadenylation by the nuclear cap binding complex (CBC). J Biol Chem 281: 4517-4522.

4. Izaurralde E, Lewis J, Gamberi C, Jarmolowski A, McGuigan C, et al. (1995) A cap-binding protein complex mediating U snRNA export. Nature 376: 709-712.

5. McKendrick L, Thompson E, Ferreira J, Morley SJ, Lewis JD (2001) Interaction of eukaryotic translation initiation factor 4G with the nuclear cap-binding complex provides a link between nuclear and cytoplasmic functions of the m(7) guanosine cap. Mol Cell Biol 21: 3632-3641.

6. Hosoda N, Kim YK, Lejeune F, Maquat LE (2005) CBP80 promotes interaction of Upf1 with Upf2 during nonsense-mediated mRNA decay in mammalian cells. Nat Struct Mol Biol 12: 893-901.

7. Ishigaki Y, Li X, Serin G, Maquat LE (2001) Evidence for a pioneer round of mRNA translation: mRNAs subject to nonsense-mediated decay in mammalian cells are bound by CBP80 and CBP20. Cell 106: 607-617.

8. Kim S, Yang JY, Xu J, Jang IC, Prigge MJ, et al. (2008) Two cap-binding proteins CBP20 and CBP80 are involved in processing primary MicroRNAs. Plant Cell Physiol 49: 1634-1644.

9. Shen V, Liu H, Liu SW, Jiao X, Kiledjian M (2008) DcpS scavenger decapping enzyme can modulate pre-mRNA splicing. RNA 14: 1132-1142.

10. Singh J, Salcius M, Liu SW, Staker BL, Mishra R, et al. (2008) DcpS as a therapeutic target for spinal muscular atrophy. ACS Chem Biol 3: 711-722.

11. Fukuto HS, Ferkey DM, Apicella AJ, Lans H, Sharmeen T, et al. (2004) G protein-coupled receptor kinase function is essential for chemosensation in C. elegans. Neuron 42: 581-593.

12. Kuwahara T, Koyama A, Koyama S, Yoshina S, Ren CH, et al. (2008) A systematic RNAi screen reveals involvement of endocytic pathway in neuronal dysfunction in alpha-synuclein transgenic C. elegans. Hum Mol Genet 17: 2997-3009.

13. Wang H, Wu LJ, Kim SS, Lee FJ, Gong B, et al. (2008) FMRP acts as a key messenger for dopamine modulation in the forebrain. Neuron 59: 634-647.

14. Piazzon N, Rage F, Schlotter F, Moine H, Branlant C, et al. (2008) In vitro and in cellulo evidences for association of the survival of motor neuron complex with the fragile X mental retardation protein. J Biol Chem 283: 5598-5610.

15. Freeman JL, Pitcher JA, Li X, Bennett V, Lefkowitz RJ (2000) alpha-Actinin is a potent regulator of G protein-coupled receptor kinase activity and substrate specificity in vitro. FEBS Lett 473: 280-284.

16. Li C, Kim K (2008) Neuropeptides. WormBook: 1-36.

17. Nelson LS, Kim K, Memmott JE, Li C (1998) FMRFamide-related gene family in the nematode, Caenorhabditis elegans. Brain Res Mol Brain Res 58: 103-111.

18. Merte J, Nichols R (2002) Drosophila melanogaster FMRFamide-containing peptides: redundant or diverse functions? Peptides 23: 209-220.

19. Cottrell GA, Green KA, Davies NW (1990) The neuropeptide Phe-Met-Arg-Phe-NH2 (FMRFamide) can activate a ligand-gated ion channel in Helix neurones. Pflugers Arch 416: 612-614.

20. Clark J, Milakovic M, Cull A, Klose MK, Mercier AJ (2008) Evidence for postsynaptic modulation of muscle contraction by a Drosophila neuropeptide. Peptides 29: 1140-1149.

21. Belardetti F, Kandel ER, Siegelbaum SA (1987) Neuronal inhibition by the peptide FMRFamide involves opening of S K+ channels. Nature 325: 153-156.

22. Sapperstein SK, Lupashin VV, Schmitt HD, Waters MG (1996) Assembly of the ER to Golgi SNARE complex requires Uso1p. J Cell Biol 132: 755-767.

23. Lupashin VV, Hamamoto S, Schekman RW (1996) Biochemical requirements for the targeting and fusion of ER-derived transport vesicles with purified yeast Golgi membranes. J Cell Biol 132: 277-289.

24. Nakajima H, Hirata A, Ogawa Y, Yonehara T, Yoda K, et al. (1991) A cytoskeleton-related gene, uso1, is required for intracellular protein transport in Saccharomyces cerevisiae. J Cell Biol 113: 245-260.

25. de Hoop M, von Poser C, Lange C, Ikonen E, Hunziker W, et al. (1995) Intracellular routing of wild-type and mutated polymeric immunoglobulin receptor in hippocampal neurons in culture. J Cell Biol 130: 1447-1459.

26. Ikonen E, Parton RG, Hunziker W, Simons K, Dotti CG (1993) Transcytosis of the polymeric immunoglobulin receptor in cultured hippocampal neurons. Curr Biol 3: 635-644.

27. Hemar A, Olivo JC, Williamson E, Saffrich R, Dotti CG (1997) Dendroaxonal transcytosis of transferrin in cultured hippocampal and sympathetic neurons. J Neurosci 17: 9026-9034.

28. Yap CC, Nokes RL, Wisco D, Anderson E, Folsch H, et al. (2008) Pathway selection to the axon depends on multiple targeting signals in NgCAM. J Cell Sci 121: 1514-1525.

29. Wisco D, Anderson ED, Chang MC, Norden C, Boiko T, et al. (2003) Uncovering multiple axonal targeting pathways in hippocampal neurons. J Cell Biol 162: 1317-1328.

30. Margeta MA, Wang GJ, Shen K (2009) Clathrin adaptor AP-1 complex excludes multiple postsynaptic receptors from axons in C. elegans. Proc Natl Acad Sci U S A 106: 1632-1637.

31. Delerive P, Chin WW, Suen CS (2002) Identification of Reverb(alpha) as a novel ROR(alpha) target gene. J Biol Chem 277: 35013-35018.

32. Guillaumond F, Dardente H, Giguere V, Cermakian N (2005) Differential control of Bmal1 circadian transcription by REV-ERB and ROR nuclear receptors. J Biol Rhythms 20: 391-403.

33. Gissendanner CR, Crossgrove K, Kraus KA, Maina CV, Sluder AE (2004) Expression and function of conserved nuclear receptor genes in Caenorhabditis elegans. Dev Biol 266: 399-416.

34. Reinking J, Lam MM, Pardee K, Sampson HM, Liu S, et al. (2005) The Drosophila nuclear receptor e75 contains heme and is gas responsive. Cell 122: 195-207.

35. Huet F, Ruiz C, Richards G (1995) Sequential gene activation by ecdysone in Drosophila melanogaster: the hierarchical equivalence of early and early late genes. Development 121: 1195-1204.

36. Segraves WA, Hogness DS (1990) The E75 ecdysone-inducible gene responsible for the 75B early puff in Drosophila encodes two new members of the steroid receptor superfamily. Genes Dev 4: 204-219.

37. Akashi M, Nishida E (2000) Involvement of the MAP kinase cascade in resetting of the mammalian circadian clock. Genes Dev 14: 645-649.

38. Szewczyk NJ, Jacobson LA (2003) Activated EGL-15 FGF receptor promotes protein degradation in muscles of Caenorhabditis elegans. EMBO J 22: 5058-5067.

39. Burdine RD, Chen EB, Kwok SF, Stern MJ (1997) egl-17 encodes an invertebrate fibroblast growth factor family member required specifically for sex myoblast migration in Caenorhabditis elegans. Proc Natl Acad Sci U S A 94: 2433-2437.

40. DeVore DL, Horvitz HR, Stern MJ (1995) An FGF receptor signaling pathway is required for the normal cell migrations of the sex myoblasts in C. elegans hermaphrodites. Cell 83: 611-620.

41. Kamikura DM, Cooper JA (2003) Lipoprotein receptors and a disabled family cytoplasmic adaptor protein regulate EGL-17/FGF export in C. elegans. Genes Dev 17: 2798-2811.

42. Kamikura DM, Cooper JA (2006) Clathrin interaction and subcellular localization of Ce-DAB-1, an adaptor for protein secretion in Caenorhabditis elegans. Traffic 7: 324-336.

43. Bruns AF, van Bergeijk J, Lorbeer C, Nolle A, Jungnickel J, et al. (2009) Fibroblast growth factor-2 regulates the stability of nuclear bodies. Proc Natl Acad Sci U S A 106: 12747-12752.

44. Shen X, Ellis RE, Sakaki K, Kaufman RJ (2005) Genetic interactions due to constitutive and inducible gene regulation mediated by the unfolded protein response in C. elegans. PLoS Genet 1: e37.

45. Wang Y, Shen J, Arenzana N, Tirasophon W, Kaufman RJ, et al. (2000) Activation of ATF6 and an ATF6 DNA binding site by the endoplasmic reticulum stress response. J Biol Chem 275: 27013-27020.

46. Yoshida H, Haze K, Yanagi H, Yura T, Mori K (1998) Identification of the cis-acting endoplasmic reticulum stress response element responsible for transcriptional induction of mammalian glucose-regulated proteins. Involvement of basic leucine zipper transcription factors. J Biol Chem 273: 33741-33749.

47. Murakami T, Hino SI, Saito A, Imaizumi K (2007) Endoplasmic reticulum stress response in dendrites of cultured primary neurons. Neuroscience 146: 1-8.

48. Gkogkas C, Middleton S, Kremer AM, Wardrope C, Hannah M, et al. (2008) VAPB interacts with and modulates the activity of ATF6. Hum Mol Genet 17: 1517-1526.

49. Nishimura AL, Mitne-Neto M, Silva HC, Richieri-Costa A, Middleton S, et al. (2004) A mutation in the vesicle-trafficking protein VAPB causes late-onset spinal muscular atrophy and amyotrophic lateral sclerosis. Am J Hum Genet 75: 822-831.

50. Bergamaschi D, Samuels Y, O'Neil NJ, Trigiante G, Crook T, et al. (2003) iASPP oncoprotein is a key inhibitor of p53 conserved from worm to human. Nat Genet 33: 162-167.

51. Bergamaschi D, Samuels Y, Sullivan A, Zvelebil M, Breyssens H, et al. (2006) iASPP preferentially binds p53 proline-rich region and modulates apoptotic function of codon 72-polymorphic p53. Nat Genet 38: 1133-1141.

52. Rotem S, Katz C, Friedler A (2007) Insights into the structure and protein-protein interactions of the pro-apoptotic protein ASPP2. Biochem Soc Trans 35: 966-969.

53. Samuels-Lev Y, O'Connor DJ, Bergamaschi D, Trigiante G, Hsieh JK, et al. (2001) ASPP proteins specifically stimulate the apoptotic function of p53. Mol Cell 8: 781-794.

54. Slee EA, Lu X (2003) The ASPP family: deciding between life and death after DNA damage. Toxicol Lett 139: 81-87.

55. Zhang XD, Wang Y, Zhang X, Han R, Wu JC, et al. (2009) p53 mediates mitochondria dysfunction-triggered autophagy activation and cell death in rat striatum. Autophagy 5.

56. Fogarty MP, McCormack RM, Noonan J, Murphy D, Gowran A, et al. (2008) A role for p53 in the beta-amyloid-mediated regulation of the lysosomal system. Neurobiol Aging.

57. Kim D, Nguyen MD, Dobbin MM, Fischer A, Sananbenesi F, et al. (2007) SIRT1 deacetylase protects against neurodegeneration in models for Alzheimer's disease and amyotrophic lateral sclerosis. EMBO J 26: 3169-3179.

58. Parlato R, Kreiner G, Erdmann G, Rieker C, Stotz S, et al. (2008) Activation of an endogenous suicide response after perturbation of rRNA synthesis leads to neurodegeneration in mice. J Neurosci 28: 12759-12764.

59. Prigent C, Glover DM, Giet R (2005) Drosophila Nek2 protein kinase knockdown leads to centrosome maturation defects while overexpression causes centrosome fragmentation and cytokinesis failure. Exp Cell Res 303: 1-13.

60. O'Regan L, Fry AM (2009) The Nek6 and Nek7 protein kinases are required for robust mitotic spindle formation and cytokinesis. Mol Cell Biol.

61. O'Regan L, Blot J, Fry AM (2007) Mitotic regulation by NIMA-related kinases. Cell Div 2: 25.

62. Barstead RJ, Kleiman L, Waterston RH (1991) Cloning, sequencing, and mapping of an alpha-actinin gene from the nematode Caenorhabditis elegans. Cell Motil Cytoskeleton 20: 69-78.

63. Virel A, Backman L (2004) Molecular evolution and structure of alpha-actinin. Mol Biol Evol 21: 1024-1031.

64. Winder SJ, Ayscough KR (2005) Actin-binding proteins. J Cell Sci 118: 651-654.

65. Gettemans J, Van Impe K, Delanote V, Hubert T, Vandekerckhove J, et al. (2005) Nuclear actin-binding proteins as modulators of gene transcription. Traffic 6: 847-857.

66. Clark KA, McElhinny AS, Beckerle MC, Gregorio CC (2002) Striated muscle cytoarchitecture: an intricate web of form and function. Annu Rev Cell Dev Biol 18: 637-706.

67. Otey CA, Carpen O (2004) Alpha-actinin revisited: a fresh look at an old player. Cell Motil Cytoskeleton 58: 104-111.

68. Peng J, Kim MJ, Cheng D, Duong DM, Gygi SP, et al. (2004) Semiquantitative proteomic analysis of rat forebrain postsynaptic density fractions by mass spectrometry. J Biol Chem 279: 21003-21011.

69. Walikonis RS, Jensen ON, Mann M, Provance DW, Jr., Mercer JA, et al. (2000) Identification of proteins in the postsynaptic density fraction by mass spectrometry. J Neurosci 20: 4069-4080.

70. Wyszynski M, Kharazia V, Shanghvi R, Rao A, Beggs AH, et al. (1998) Differential regional expression and ultrastructural localization of alpha-actinin-2, a putative NMDA receptor-anchoring protein, in rat brain. J Neurosci 18: 1383-1392.

71. Hunt-Newbury R, Viveiros R, Johnsen R, Mah A, Anastas D, et al. (2007) High-throughput in vivo analysis of gene expression in Caenorhabditis elegans. PLoS Biol 5: e237.

72. McKay SJ, Johnsen R, Khattra J, Asano J, Baillie DL, et al. (2003) Gene expression profiling of cells, tissues, and developmental stages of the nematode C. elegans. Cold Spring Harb Symp Quant Biol 68: 159-169.

73. Pan S, Wang R, Zhou X, He G, Koomen J, et al. (2006) Involvement of the conserved adaptor protein Alix in actin cytoskeleton assembly. J Biol Chem 281: 34640-34650.

74. Huttelmaier S, Illenberger S, Grosheva I, Rudiger M, Singer RH, et al. (2001) Raver1, a dual compartment protein, is a ligand for PTB/hnRNPI and microfilament attachment proteins. J Cell Biol 155: 775-786.

75. Li Q, Montalbetti N, Shen PY, Dai XQ, Cheeseman CI, et al. (2005) Alpha-actinin associates with polycystin-2 and regulates its channel activity. Hum Mol Genet 14: 1587-1603.

76. Sadeghi A, Doyle AD, Johnson BD (2002) Regulation of the cardiac L-type Ca2+ channel by the actin-binding proteins alpha-actinin and dystrophin. Am J Physiol Cell Physiol 282: C1502-1511.

77. Maruoka ND, Steele DF, Au BP, Dan P, Zhang X, et al. (2000) alpha-actinin-2 couples to cardiac Kv1.5 channels, regulating current density and channel localization in HEK cells. FEBS Lett 473: 188-194.

78. Krupp JJ, Vissel B, Thomas CG, Heinemann SF, Westbrook GL (1999) Interactions of calmodulin and alpha-actinin with the NR1 subunit modulate Ca2+-dependent inactivation of NMDA receptors. J Neurosci 19: 1165-1178.

79. Allison DW, Gelfand VI, Spector I, Craig AM (1998) Role of actin in anchoring postsynaptic receptors in cultured hippocampal neurons: differential attachment of NMDA versus AMPA receptors. J Neurosci 18: 2423-2436.

80. Rajendra TK, Gonsalvez GB, Walker MP, Shpargel KB, Salz HK, et al. (2007) A Drosophila melanogaster model of spinal muscular atrophy reveals a function for SMN in striated muscle. J Cell Biol 176: 831-841.

81. Chen HW, Marinissen MJ, Oh SW, Chen X, Melnick M, et al. (2002) CKA, a novel multidomain protein, regulates the JUN N-terminal kinase signal transduction pathway in Drosophila. Mol Cell Biol 22: 1792-1803.

82. Benoist M, Gaillard S, Castets F (2006) The striatin family: a new signaling platform in dendritic spines. J Physiol Paris 99: 146-153.

83. Castets F, Bartoli M, Barnier JV, Baillat G, Salin P, et al. (1996) A novel calmodulin-binding protein, belonging to the WD-repeat family, is localized in dendrites of a subset of CNS neurons. J Cell Biol 134: 1051-1062.

84. Lau CK, Bachorik JL, Dreyfuss G (2009) Gemin5-snRNA interaction reveals an RNA binding function for WD repeat domains. Nat Struct Mol Biol.

85. Murphey RK, Caruccio PC, Getzinger M, Westgate PJ, Phillis RW (1999) Dynein-dynactin function and sensory axon growth during Drosophila metamorphosis: A role for retrograde motors. Dev Biol 209: 86-97.

86. Bullock SL, Nicol A, Gross SP, Zicha D (2006) Guidance of bidirectional motor complexes by mRNA cargoes through control of dynein number and activity. Curr Biol 16: 1447-1452.

87. Ghosh-Roy A, Desai BS, Ray K (2005) Dynein light chain 1 regulates dynamin-mediated F-actin assembly during sperm individualization in Drosophila. Mol Biol Cell 16: 3107-3116.

88. Kim H, Ling SC, Rogers GC, Kural C, Selvin PR, et al. (2007) Microtubule binding by dynactin is required for microtubule organization but not cargo transport. J Cell Biol 176: 641-651.

89. McCabe BD, Marques G, Haghighi AP, Fetter RD, Crotty ML, et al. (2003) The BMP homolog Gbb provides a retrograde signal that regulates synaptic growth at the Drosophila neuromuscular junction. Neuron 39: 241-254.

90. Chang HC, Dimlich DN, Yokokura T, Mukherjee A, Kankel MW, et al. (2008) Modeling spinal muscular atrophy in Drosophila. PLoS ONE 3: e3209.

91. Chevalier-Larsen E, Holzbaur EL (2006) Axonal transport and neurodegenerative disease. Biochim Biophys Acta 1762: 1094-1108.

92. Batlevi Y, Martin DN, Pandey UB, Simon CR, Powers CM, et al. Dynein light chain 1 is required for autophagy, protein clearance, and cell death in Drosophila. Proc Natl Acad Sci U S A 107: 742-747.

93. Keen JE, Khawaled R, Farrens DL, Neelands T, Rivard A, et al. (1999) Domains responsible for constitutive and Ca(2+)-dependent interactions between calmodulin and small conductance Ca(2+)-activated potassium channels. J Neurosci 19: 8830-8838.

94. Schumacher MA, Rivard AF, Bachinger HP, Adelman JP (2001) Structure of the gating domain of a Ca2+-activated K+ channel complexed with Ca2+/calmodulin. Nature 410: 1120-1124.

95. Xia XM, Fakler B, Rivard A, Wayman G, Johnson-Pais T, et al. (1998) Mechanism of calcium gating in small-conductance calcium-activated potassium channels. Nature 395: 503-507.

96. Bond CT, Herson PS, Strassmaier T, Hammond R, Stackman R, et al. (2004) Small conductance Ca2+-activated K+ channel knock-out mice reveal the identity of calcium-dependent afterhyperpolarization currents. J Neurosci 24: 5301-5306.

97. Stocker M, Krause M, Pedarzani P (1999) An apamin-sensitive Ca2+-activated K+ current in hippocampal pyramidal neurons. Proc Natl Acad Sci U S A 96: 4662-4667.

98. Blatz AL, Magleby KL (1986) Single apamin-blocked Ca-activated K+ channels of small conductance in cultured rat skeletal muscle. Nature 323: 718-720.

99. Abel HJ, Lee JC, Callaway JC, Foehring RC (2004) Relationships between intracellular calcium and afterhyperpolarizations in neocortical pyramidal neurons. J Neurophysiol 91: 324-335.

100. Cai X, Liang CW, Muralidharan S, Kao JP, Tang CM, et al. (2004) Unique roles of SK and Kv4.2 potassium channels in dendritic integration. Neuron 44: 351-364.

101. Shakkottai VG, Chou CH, Oddo S, Sailer CA, Knaus HG, et al. (2004) Enhanced neuronal excitability in the absence of neurodegeneration induces cerebellar ataxia. J Clin Invest 113: 582-590.

102. McKee P, Fuller GN, Stevens DL (1994) Riluzole in amyotrophic lateral sclerosis. N Engl J Med 331: 272; author reply 273-274.

103. Bensimon G, Lacomblez L, Meininger V (1994) A controlled trial of riluzole in amyotrophic lateral sclerosis. ALS/Riluzole Study Group. N Engl J Med 330: 585-591.

104. Couratier P, Sindou P, Esclaire F, Louvel E, Hugon J (1994) Neuroprotective effects of riluzole in ALS CSF toxicity. Neuroreport 5: 1012-1014.

105. Cao YJ, Dreixler JC, Couey JJ, Houamed KM (2002) Modulation of recombinant and native neuronal SK channels by the neuroprotective drug riluzole. Eur J Pharmacol 449: 47-54.

106. Haddad H, Cifuentes-Diaz C, Miroglio A, Roblot N, Joshi V, et al. (2003) Riluzole attenuates spinal muscular atrophy disease progression in a mouse model. Muscle Nerve 28: 432-437.

107. Russman BS, Iannaccone ST, Samaha FJ (2003) A phase 1 trial of riluzole in spinal muscular atrophy. Arch Neurol 60: 1601-1603.

108. Bosboom WM, Vrancken AF, van den Berg LH, Wokke JH, Iannaccone ST (2009) Drug treatment for spinal muscular atrophy type I. Cochrane Database Syst Rev: CD006281.

109. Turner BJ, Parkinson NJ, Davies KE, Talbot K (2009) Survival motor neuron deficiency enhances progression in an amyotrophic lateral sclerosis mouse model. Neurobiol Dis 34: 511-517.

110. Lu L, Zhang Q, Timofeyev V, Zhang Z, Young JN, et al. (2007) Molecular coupling of a Ca2+-activated K+ channel to L-type Ca2+ channels via alpha-actinin2. Circ Res 100: 112-120.

111. Koelle MR, Talbot WS, Segraves WA, Bender MT, Cherbas P, et al. (1991) The Drosophila EcR gene encodes an ecdysone receptor, a new member of the steroid receptor superfamily. Cell 67: 59-77.

112. Yao TP, Forman BM, Jiang Z, Cherbas L, Chen JD, et al. (1993) Functional ecdysone receptor is the product of EcR and Ultraspiracle genes. Nature 366: 476-479.

113. Calderon F, Kim HY (2007) Role of RXR in neurite outgrowth induced by docosahexaenoic acid. Prostaglandins Leukot Essent Fatty Acids 77: 227-232.

114. Hoover LL, Burton EG, O'Neill ML, Brooks BA, Sreedharan S, et al. (2008) Retinoids regulate TGFbeta signaling at the level of Smad2 phosphorylation and nuclear accumulation. Biochim Biophys Acta 1783: 2279-2286.
